# Research Progress on the Protective Effect of Green Tea Polyphenol (-)-Epigallocatechin-3-Gallate (EGCG) on the Liver

**DOI:** 10.3390/nu17071101

**Published:** 2025-03-21

**Authors:** Fang Zhou, Sengwen Deng, Yong Luo, Zhonghua Liu, Changwei Liu

**Affiliations:** 1 School of Chemistry and Environmental Sciences, Xiangnan University, Chenzhou 423000, China; fangzhou@xnu.edu.cn; 2 School of Life and Health Sciences, Hunan University of Science and Technology, Xiangtan 411201, China; sengdengden@163.com (S.D.); lcvv666@163.com (C.L.); 3 Key Laboratory of Tea Science of Ministry of Education, Hunan Agricultural University, Changsha 410128, China; zhonghua-liu-ms@hunau.edu.cn

**Keywords:** liver-related disease, EGCG, viral hepatitis, autoimmune hepatitis, fatty liver disease, hepatocellular carcinoma

## Abstract

The liver, as the primary metabolic organ, is susceptible to an array of factors that can harm liver cells and give rise to different liver diseases. Epigallocatechin gallate (EGCG), a natural compound found in green tea, exerts numerous beneficial effects on the human body. Notably, EGCG displays antioxidative, antibacterial, antiviral, anti-inflammatory, and anti-tumor properties. This review specifically highlights the pivotal role of EGCG in liver-related diseases, focusing on viral hepatitis, autoimmune hepatitis, fatty liver disease, and hepatocellular carcinoma. EGCG not only inhibits the entry and replication of hepatitis B and C viruses within hepatocytes, but also mitigates hepatocytic damage caused by hepatitis-induced inflammation. Furthermore, EGCG exhibits significant therapeutic potential against hepatocellular carcinoma. Combinatorial use of EGCG and anti-hepatocellular carcinoma drugs enhances the sensitivity of drug-resistant cancer cells to chemotherapeutic agents, leading to improved therapeutic outcomes. Thus, the combination of EGCG and anti-hepatocellular carcinoma drugs holds promise as an effective approach for treating drug-resistant hepatocellular carcinoma. In conclusion, EGCG possesses hepatoprotective properties against various forms of liver damage and emerges as a potential drug candidate for liver diseases.

## 1. Introduction

The liver, situated in the abdominal region, serves as the largest digestive gland in the body. It functions as the primary metabolic organ, playing a vital role in the maintenance of physiological stability. The liver, in turn, is influenced by various external and internal factors, some of which damage liver cells and cause liver diseases. Viral infections, including hepatitis B virus (HBV) and hepatitis C virus (HCV) infections, are a common cause of liver diseases. They can cause high levels of liver inflammation, and persistent viral infection can lead to fibrosis, cirrhosis, and even liver cancer, causing serious health concerns [1]. Furthermore, improvements in the standard of living and the concurrent increase in poor lifestyle habits and lack of sufficient exercise have contributed to a rapid increase in the prevalence of obesity. Large amounts of fat can accumulate in liver cells, leading to the development of a fatty liver. This affects the normal physiological functions of the liver and induces an inflammatory response that can cause further liver damage [2,3]. 

Liver-related diseases are a serious threat to human health. Hepatic autoimmune hepatitis, a condition typically observed in women, involves chronic progressive liver inflammation caused by an autoimmune response. The specific pathogenesis of this disease is unknown, but patients with severe hepatic autoimmune hepatitis can rapidly develop cirrhosis and liver failure [4]. Hepatocellular carcinoma is one of the most fatal malignancies in the world, and its prevalence has been increasing every year. Moreover, viral hepatitis and steatohepatitis may eventually develop into hepatocellular carcinoma if not treated in a timely manner [5]. Therefore, the identification of food supplements or drugs that can help prevent and treat liver diseases is an important research focus currently.

Tea is one of the most popular beverages in the world. China is considered the birthplace of tea. It was the first country in the world to grow the tea plant [*Camellia sinensis* (L.) O. Kuntze] and has cultivated tea plants for thousands of years [6,7]. Based on their production processes, teas can be categorized into six types: green tea, black tea, white tea, dark tea, yellow tea, and oolong tea. These teas vary in terms of color, aroma, taste, and the range of beneficial effects they offer for human health. These effects are primarily attributed to the distinctive chemical compounds generated in tea leaves during the different production processes [8]. Green tea, widely consumed as a daily beverage, accounts for approximately 20% of global tea consumption, with even higher rates in China. Its production involves the stages of fixing, rolling, and drying, wherein high temperatures inactivate endogenous enzymes and optimize the preservation of primary and secondary metabolites in tea leaves. Among the different metabolites, polyphenols are present at high levels in tea leaves, accounting for about 18–36% of their dry weight. Tea polyphenols mainly include catechins, flavonoids and flavonoid glycosides, anthocyanins, phenolic acids, and condensed phenolic acids. Among these, catechins are the main active products, accounting for about 12–24% of the dry weight of tea leaves. Catechins are a class of flavanols with a 2-phenyl-dihydropyran structure. They are synthesized in the tea tree via three steps: the mangiferic acid pathway, phenyl propane pathway, and flavonoid pathway [9]. There are four main types of catechin: epicatechin (EC), epigallocatechin (EGC), epicatechin gallate (ECG), and epigallocatechin gallate (EGCG). Of these, EGCG shows the highest levels, accounting for 50–80% of the total catechins in green tea [10]. Over the past 20 years, a large number of in vitro and in vivo studies and epidemiological data have shown that EGCG has useful antiviral, antibacterial, antioxidant, hypoglycemic, and hypolipidemic functions. Furthermore, EGCG can also prevent cardiovascular and cerebrovascular diseases and protect the liver, kidneys, and nervous system, making it an important health-boosting agent [11,12,13]. Their excellent antioxidant capacity and physiological activity indicate that catechins are ideal raw materials for the development of daily supplements, functional foods, and medicines.

This comprehensive review assesses how EGCG modulates the regulatory effects in various liver diseases, including viral hepatitis, autoimmune hepatitis, fatty liver disease, and hepatocellular carcinoma. Furthermore, the paper summarizes the promising therapeutic effects of combining EGCG with anti-tumor compounds in the treatment of liver cancer. Additionally, an in-depth analysis of the synergistic effects of EGCG with anti-tumor compounds further bridges the gap between mechanistic understanding and therapeutic innovation in liver cancer treatment. By systematically integrating molecular-level evidence, this review positions EGCG as a multifaceted candidate that contributes to our understanding of liver pathophysiology and advances therapeutic strategies.

## 2. EGCG: An Overview

### 2.1. Physicochemical Characteristics of EGCG and Its Bioavailability

**Figure 1:**
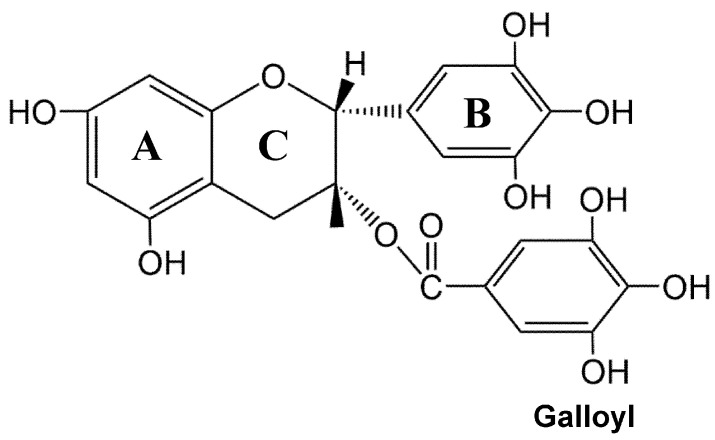
Chemical structure of (-)-epigallocatechin-3-gallate.

EGCG, an active catechin, shares a similar chemical structure with other catechins. Its chemical composition consists of a skeleton comprising three fundamental ring nuclei (A, B, and C) and a galloyl residue. The A ring bears m-dihydroxyl groups at positions 5 and 7; the B ring possesses three hydroxyl groups at positions 3, 4, and 5; and the C ring is a pyran ring (Figure 1). EGCG exhibits a higher number of phenolic hydroxyl groups compared to other catechins found in green tea, conferring potent antioxidant properties, enabling it to effectively scavenge free radicals, eliminate reactive oxygen species (ROS), and maintain normal cellular functions [14]. The polyphenolic structure of EGCG imparts distinct chemical characteristics. Notably, EGCG has the ability to interact with proteins, polysaccharides, and alkaloids, forming complexes with various metal ions. Furthermore, its robust reduction and free radical capture properties contribute to its substantial antioxidant biological activity [15]. The diverse physiological activities of EGCG arise from these fundamental chemical properties [10]. 

Upon oral administration, EGCG undergoes primary metabolism in the stomach and small intestine. Following absorption by the lining of the small intestine, EGCG is metabolized by the liver, transported through the circulatory system to various tissues and organs, and ultimately excreted in urine or feces [16]. Administering EGCG orally to rodents only results in peak plasma concentration in the range of nM or low micromolar [17]. Pharmacokinetic studies in rats have demonstrated that the bioavailability of EGCG after oral administration is merely 1%. However, this value can increase to 14% when decaffeinated catechins are administered intragastrically [18,19]. Merely 0.1–1.1% of the EGCG dose reaches systemic circulation [20]. In fasting rats, after a single oral administration of 56mg of EGCG, the maximum plasma concentration of EGCG was observed at 1047 ng/mL (approximately 0.012% of the EGCG intake), while fasting individuals exhibited a maximum plasma concentration of 156 ng/mL (about 0.32% of the EGCG intake) [21]. Consequently, the bioavailability of EGCG is poor, as only a small proportion of the orally administered EGCG is absorbed into the blood, with the possibility of efflux phenomena [22]. Research suggests that a minimum concentration of 10 μM is required to achieve the relevant therapeutic effects of EGCG [23]. Therefore, the low oral bioavailability may serve as the primary obstacle in elucidating the efficacy of bioactive substances and the significant individual differences observed in clinical trials, consequently hindering further development and utilization. In mice, the bioavailability of free EGCG has been reported as 12.4%. The absolute bioavailability of total EGCG, after treatment with glucuronidase and sulfate esterase, can reach up to 26.5%, highlighting the potential for delivery modes and modification treatments to substantially enhance EGCG’s bioavailability [24]. This effect is closely associated with the inherent properties of EGCG. Lipinski’s Rule of Five posits that bioavailability is influenced by molecular size, apparent size (hydration shell formation), and polarity [25]. EGCG, with a relative molecular mass of 458.38 and eight phenolic hydroxyl groups, exhibits comparatively lower bioavailability than EC (relative molecular mass of 290.75, with five phenolic hydroxyl groups). Additionally, the extensive hydration shell formed by the hydrogen bond between EGCG and water molecules diminishes its absorption within the body. However, these factors can enhance the ability of EGCG to bind to proteins and nucleic acids, ultimately augmenting its active effect. 

In conclusion, the bioactivity of EGCG is limited after oral administration. Furthermore, its absorption and metabolic processing in the body are influenced by a range of internal factors such as intestinal microbial catabolism, as well as external factors including the food matrix and intake. However, the bioavailability of EGCG can be enhanced through chemical modifications. Additionally, modifications in the mode of administration and combination with adjuvant drugs have the potential to improve EGCG’s bioavailability.

### 2.2. Safety of EGCG

EGCG, a natural plant product, has received considerable attention regarding its safety profile. A study on the safety of EGCG investigated the genetic, acute, and short-term toxicity effects, establishing a no-observed-adverse-effect level (NOAEL) of 500 mg/kg/day. The LD_50_ value of orally administered EGCG in rats ranges from 186.8 mg/kg (considered safe) to 1868 mg/kg (indicating potential toxic effects and animal morbidity and mortality) [26]. In humans, the NOAEL is reported to be 600 mg per day. An acceptable daily intake of EGCG for adults weighing 70 kg has been reported as 322 mg [27]. A small, randomized placebo-controlled trial involving eight healthy volunteers per group demonstrated the safety and tolerability of EGCG and catechin mixtures at a dose of 800 mg/day for four weeks [28]. In a randomized placebo-controlled clinical trial that included 1075 postmenopausal women, 538 women in the green tea extract (GTE) group received a supplementation of 1315 mg of catechins per day (including 843 mg of EGCG), while 537 individuals were assigned to the placebo group. With a duration of 1 year, 957 subjects completed the trial, and favorable outcomes were observed in the GTE supplementation group without any adverse effects associated with EGCG [29]. Based on available toxicological and safety data, a statistical analysis on the safety of EGCG confirmed that a dosage of 338 mg of EGCG in solid form is safe. Furthermore, no adverse effects were reported for adults consuming 704 mg of EGCG daily through tea consumption, possibly because other substances in tea, such as theanine, can counteract the side effects of high doses of EGCG [30]. However, an excessive dose of EGCG can lead to toxic side effects, including hepatotoxicity, insomnia, nausea, abdominal pain, and diarrhea [31,32]. Mice receiving a single injection of 1500 mg/kg EGCG per day or two separate doses of 750 mg/kg EGCG exhibited elevated levels of glutamate transferase, liver toxicity, and severe necrosis. These mice also demonstrated increased oxidative stress in the liver, including heightened lipid peroxidation, elevated plasma 8-isoprostane levels, and increased expression of hepatic metallothionein and histone H2Ax [33]. These findings align with reports suggesting that high doses of EGCG can promote oxidation instead of antioxidant effects, resulting in reactive oxygen species (ROS) production, disruption of mitochondrial membrane potential, varied types of cell damage, and liver injury [28]. However, according to the USDA flavonoid database, an average cup of green tea derived from 1 g of tea leaves soaked in 100 mL of boiling water contains approximately 126.6 mg of total catechins and 77.8 mg of EGCG. The average dose of EGCG exposure in adults from brewed green tea is 321 mg daily [34]. Studies have shown that approximately 400 mg to 600 mg of catechins per day (approximately equivalent to four to six cups of green tea per day) or 115 mg to 300 mg of EGCG (approximately equivalent to two to five cups of green tea per day) may have health benefits for healthy adults [35]. Therefore, even if you regularly drink 8–10 cups of green tea per day, it will not have any negative effects on human health. Nonetheless, when taking EGCG in solid form as a dietary supplement or medication, supervision by a healthcare professional is recommended.

## 3. Effect of EGCG in Liver Diseases

Several internal and external factors can contribute to liver-related diseases. Here, we review the therapeutic roles of EGCG in viral hepatitis, autoimmune hepatitis, fatty liver, and hepatocellular carcinoma, clarifying its effects and the specific underlying mechanisms (Table 1).

### 3.1. Viral Hepatitis

Viruses play a significant role in the development of hepatitis, with HBV and HCV being the primary causative agents. HBV and HCV demonstrate high infectivity and rank among the most prevalent viral causes of hepatitis in the human population, globally.

Hepatitis B virus (HBV), a member of the hepatoviral family, is responsible for a global infection rate of approximately 350 million people, establishing it as the most pervasive hepatitis virus [65]. The HBV genome resides within a nucleocapsid, comprising a 3.2 kb relaxed circular partial double-stranded DNA (RC-DNA), and is enveloped by three types of surface antigen (S, M, and L), each with distinct functions [66,67,68]. While arising from a shared open reading frame, these surface proteins possess variations in their *N*-terminal flanking sequences. For instance, the M-type surface antigen includes a pre-S2 structural domain consisting of 55 amino acids [69]. In contrast, the L-type surface antigen has a pre-S1 structural domain, an extension of the pre-S2 structural domain, comprising an additional 108 or 119 amino acids, depending on the viral genotype [69], and plays a pivotal role in viral entry [70,71]. Studies underscore the efficacy of green tea extract (GTE) in reducing HBV virions in infected cells, with a prominent component, EGCG, demonstrating superior effectiveness in inhibiting HBV infection and replication [36]. EGCG can impede HBV entry into host cells, achieving up to an 80% inhibition at a concentration of 50 μM, with this effect observed across various HBV genotypes (type A to type D). Furthermore, EGCG induces endocytosis and degradation of the HBV receptor, sodium-taurocholate cotransporting polypeptide (NTCP), serving as a molecular mechanism hindering HBV’s entry into host cells [37]. Another study substantiates the inhibitory effect of EGCG on HBV infection, leading to reduced serum levels of HBV DNA and surface antigens [38]. These findings unambiguously affirm the beneficial effects of EGCG in inhibiting HBV infection and its pivotal role in suppressing HBV replication.

EGCG has demonstrated its ability to inhibit HBV replication in HepG2.117 cells by impeding the formation of HBV replicative intermediates during DNA synthesis, which ultimately reduces the production of covalently closed circular DNA [39]. Treatment with EGCG has also exhibited reductions in HBsAg and HBeAg levels in the supernatant of Hep3B2.1-7 cells, showing consistent findings with decreased HBV DNA replication observed in HepG2 2.2.15 cells [40,41]. In the context of HepG2.2.15 cells, EGCG inhibits DNA replication by activating the ERK1/2 signaling pathway, subsequently leading to the downregulation of hepatocyte nuclear factor 4α (HNF4α) mRNA and protein expressions. Thus, EGCG inhibits HBV gene expression and replication. However, the ERK1/2 inhibitor PD98059 can reverse this process [42]. Additionally, EGCG modulates the regulation of HBV antigens by interacting with FXRα, which, in turn, regulates HBV antigens and activates Enhll core promoter activity through the FXRα/RXRα axis [43]. Autophagy plays a pivotal role in HBV replication, where autophagosomes encapsulate incorrectly folded proteins or substances, followed by fusion with lysosomes to form autolysosomes. Consequently, the cellular components enclosed within autolysosomes are degraded, providing the cell with reusable elements. In HBV-infected cells, the virus promotes its own replication by enhancing intracellular autophagosome formation [72,73,74]. Nevertheless, HBV-induced autophagy is incomplete, as P62, a protein associated with autophagy, remains undegraded. Moreover, HBV reduces the acidification rate and degradative activity of lysosomes. In contrast, EGCG stimulates complete cellular autophagy, resulting in P62 degradation by enhancing the acidification rate and lysosomal degradative activity. This alternative pathway elucidates how EGCG inhibits HBV replication [75].

Hepatitis C is characterized by liver inflammation induced by infection with the hepatitis C virus (HCV), often resulting in severe liver damage. The World Health Organization estimates that approximately 2.5% of the global population, equating to over 177.5 million individuals, are chronically infected with HCV and are at risk of developing liver diseases, including liver cancer [76,77]. Consequently, the development of targeted drugs for HCV is of paramount importance. The current standard therapy, consisting of pegylated interferon (IFN)-α and ribavirin, has limited efficacy and significant adverse effects [78]. The use of antiviral drugs alone poses the risk of viral drug resistance and can prove highly costly [79]. Therefore, the identification of HCV target molecules that offer minimal adverse effects and are cost-effective remains crucial for effective HCV management. Through an RNA aptamer sensor system, EGCG exhibited high affinity and inhibition efficiency against the HCV-encoded NS5B viral protein, suggesting its potential as a candidate for HCV treatment [44]. Employing a cell-based microplate colorimetric screening system using HCV-JFH1 virus culture, EGCG demonstrated remarkably potent antiviral activity, inhibiting HCV entry into cells [80]. At the cellular level, EGCG exhibited significant anti-HCV effects. In an HCV-infected systems model, EGCG prevented HCV particles from attaching to the cell surface, thus impeding viral entry. Corresponding results were observed in studies conducted with primary hepatocytes [45,46]. The half-maximal inhibitory concentration (IC50) of EGCG against HCV infection was measured to be 17.9 μM in an infection model. Moreover, EGCG effectively inhibited HCV mRNA and protein expression in Huh7.5.1 cells infected with the JFH1 virus. Continuous treatment with 50 and 25 μM EGCG for 2–5 generations led to rapid clearance of the HCV virus from host cells, highlighting the potential of EGCG as a candidate for HCV infection prevention.

MicroRNAs are a class of non-coding RNAs that play a pivotal role in gene regulation. EGCG has been found to have the potential to inhibit HCV activity by upregulating miR-194, a microRNA that targets CD81, one of the main receptors of HCV. Consequently, EGCG can downregulate the expression of CD81 through miR-194, effectively preventing HCV from entering cells [79]. Additionally, EGCG’s mediation leads to the downregulation of CD81 by miR-548, which is another key intermediate. In Huh7 cells, EGCG upregulates miR-548, which targets CD81, ultimately leading to diminished mRNA and protein levels of CD81 and inhibition of HCV infection [81]. Furthermore, EGCG enhances cellular innate immunity during HCV infection. When HCV infection takes place, double-stranded RNA produced by HCV induces the intracellular expression of Toll-like receptor 3 (TLR3), RIG-I, and certain IFN-stimulated genes (ISGs) that have antiviral properties. Although EGCG alone has an insignificant effect on TLR3 and RIG-I signaling, it significantly augments the expression of IFN-λ1, TLR3, RIG-I, and antiviral ISGs induced by HCV double-stranded RNA in hepatocytes. This enhancement of innate immunity protects hepatocytes against HCV infection [47].

While the precise mechanism by which EGCG inhibits viral infection remains unclear, existing evidence suggests that catechins and their derivatives possess potential in combating hepatitis B and C. These compounds stand as viable targets for protection and resistance against potential hepatitis infections. Additionally, viral hepatitis can be caused by hepatitis A, hepatitis D, and hepatitis E viruses. However, currently, there is a lack of research reporting the effects of catechins on these viruses, and therefore, further studies are warranted. In addition, the present research predominantly concentrates on cellular and animal experiments, with a limited presence of clinical research. This poses a significant challenge for translating findings from cell and animal studies to clinical applications.

### 3.2. Autoimmune Hepatitis

Autoimmune hepatitis is a chronic self-sustaining inflammatory disease. This disease has a female sex preponderance and can occur at all ages. It typically starts with an autoimmune hepatitis attack and then progresses to liver fibrosis, liver cancer, or even death. Immunosuppression and liver transplantation are effective in treating autoimmune hepatitis. The use of endocorticosteroids alone or in combination with endocorticosteroids and azathioprine has been shown to be effective in prolonging patient survival, but challenges owing to poor outcomes in some patients remain [82]. EGCG has anti-inflammatory activity, and in LPS-induced macrophages, EGCG significantly reduces the mRNA levels of iNOS and attenuates the LPS-induced inflammatory response [83]. In addition, EGCG inhibits LPS-induced TNF-α production in RAW264.7 macrophages in a dose-dependent manner. Studies have revealed that EGCG can inhibit the transcription of TNF-α by downregulating NF-κB activity. EGCG has also been shown to downregulate LPS-induced TNF-α expression in BALB/c mice, reducing serum TNF-α levels by nearly 80% and completely inhibiting LPS-induced liver injury-related lethality [48]. Therefore, EGCG may also have an important therapeutic role in autoimmune hepatitis.

An intravenous injection of lectin with concanavalin A (ConA) is commonly used to model hepatitis in mice. Unlike in other hepatitis models, in the ConA model, liver injury is mainly induced by the activation and recruitment of T cells to the liver, resulting in a highly inflammatory environment. This model is also used for studying autoimmune hepatitis. In one study, mouse models of ConA-induced acute liver injury were fed 5 mg/kg of EGCG twice daily for 10 days. The next post-ConA treatment assay showed that EGCG attenuated ConA-induced liver injury, inhibited alanine aminotransferase (ALT) levels in mouse plasma, and reduced inflammatory cell infiltration and hepatocyte apoptosis in the liver. The mRNA and protein levels of TNF-α and IFN-γ were also effectively downregulated in the livers of mice treated with EGCG; moreover, iNOS was downregulated and NO production was inhibited. In addition, the mRNA expression of IP-10 and MIP-1α was significantly reduced [84]. It has been speculated that EGCG inhibits the ConA-induced activation of NF-κB; downregulates the expressions of TLR2, TLR4, and TLR9; inhibits the production of TNF-α, IFN-γ, IL-4, and IL-6 in the mouse liver; and also reduces malondialdehyde (MDA) levels. Further, it allows the recovery of glutathione (GSH) and superoxide dismutase (SOD) activity [85]. In another study, EGCG was found to inhibit the expression of the ConA-induced inflammatory cytokines TNF-α, IFN-γ, IL-1β, and IL-6 and thus suppress the immune response. EGCG can also reduce the binding of BNIP3 to BCL2 by inhibiting the IL-6/JAKs/STAT3/BNIP3 axis, thereby stabilizing the BCL-2/Beclin complex, blocking Beclin-1 release, inhibiting hepatocyte autophagy, and enhancing anti-apoptotic capacity in hepatocytes [49]. Together, this evidence suggests that EGCG could be a suitable candidate for the treatment of autoimmune hepatitis.

### 3.3. Fatty Liver Disease

**Figure 2:**
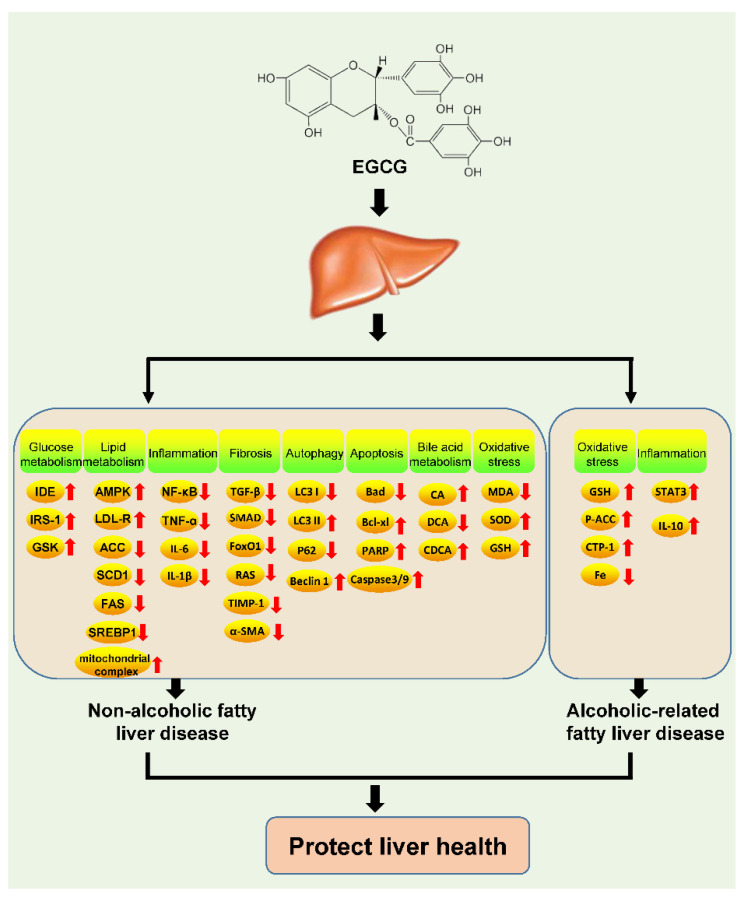
Mechanism underlying the effect of EGCG on fatty liver disease (upward arrow: increase; downward arrow: decrease).

Fatty liver, a clinicopathological syndrome caused by the accumulation of fat in liver cells, is a common liver disease in humans. This disease can be caused by a variety of factors, and its prevalence is increasing with the improvement in standards of living around the world. Fatty liver can cause a series of diseases, affecting the digestive and cardiovascular systems. It is a serious threat to public health, especially given the rise in its incidence with the increasing global rates of obesity. Epidemiological studies show that the global prevalence of fatty liver in adults has exceeded 25% [86]. If fatty liver is not controlled, it can cause liver fibrosis, cirrhosis, and even liver cancer [87]. Clinically, fatty liver disease can be classified into non-alcoholic fatty liver disease (NAFLD) and alcohol-related fatty liver disease (AFLD), based on the cause of the disease. There is currently no specific treatment for fatty liver disease, although studies have found that EGCG has a good therapeutic effect on both NAFLD and AFLD (Figure 2).

### 3.4. Non-Alcoholic Fatty Liver Disease

NAFLD is a common liver disease. It is characterized by the accumulation of large amounts of triglycerides (TGs) in hepatocytes in patients with no significant history of alcohol consumption or abuse. NAFLD is a metabolic disease, and its incidence has increased with improvements in living standards, changes in dietary habits, increase in stress levels, and the rise of other related diseases such as diabetes, hyperlipidemia, and metabolic syndrome. Therefore, NAFLD has become a major public health problem. If timely interventions are not made, NAFLD can progress to non-alcoholic steatohepatitis, cirrhosis, and hepatocellular carcinoma, and in some cases, liver transplantation may become necessary [88,89,90]. However, adequate treatments for NAFLD are currently unavailable. Although weight loss and exercise can effectively control the development of fatty liver, meeting these goals is challenging. Therefore, a combination of lifestyle changes and complementary medication appears to be necessary [91].

Many natural compounds present in food items can improve NAFLD and are considered potential candidates for NAFLD prevention and treatment. As a natural product with anti-inflammatory and antioxidant activities, EGCG has shown excellent efficacy in the prevention and treatment of NAFLD [91]. The effect of EGCG on NAFLD is multi-modal and involves multiple mechanisms. Fatty liver is caused by the storage of a large amount of fat that cannot be metabolized in time and thus causes metabolic imbalance. Insulin plays a pivotal role in glucose metabolism by promoting glucose catabolism and gluconeogenesis. However, tissues that are chronically exposed to high levels of insulin become insulin resistant, which negatively affects glucose metabolism [92]. Insulin resistance is strongly associated with NAFLD and is a risk factor for the disease [93,94]. In a high-fat diet-induced NAFLD model, EGCG was found to promote insulin clearance in hepatocytes and improve cellular sensitivity to insulin by upregulating the expression of insulin-degrading enzyme (IDE). In addition, EGCG was found to promote the expression of the insulin receptor IRS-1 and glycogen synthase kinase (GSK), enhancing glucose metabolism and reducing the incidence of NAFLD [50,95].

Disorders of lipid metabolism are responsible for NAFLD. In high-fat-induced hepatic steatosis, EGCG supplementation can significantly reduce TG and cholesterol levels in the liver. This could be because EGCG promotes the oxidative degradation of free fatty acids by activating AMPK while increasing the expression of low-density lipoprotein receptor (LDL-R) and promoting cholesterol metabolism [51,52,96]. EGCG can also promote fatty acid metabolism in hepatocytes by suppressing the expression of the sterol regulatory element-binding protein-1 (SREBP-1) gene and downregulating its downstream target genes ACC, FAS, and SCD1 [53,54]. EGCG also increases the oxidation of long-chain fatty acids by increasing the activity of the mitochondrial complex, thus halting NAFLD progression [55]. Fat accumulation in hepatocytes can cause a high level of infiltration by inflammatory cells, which secrete several inflammatory cytokines and generate an inflammatory microenvironment, leading to liver fibrosis. In the liver, EGCG can inhibit the NF-κB signaling pathway; downregulate the mRNA expression of TNF-α, IL-6, IL-1β, and monocyte chemoattractant protein-1 (MCP-1); reduce the secretion of inflammatory cytokines; and induce the conversion of macrophages from the M1 to the M2 type. Accordingly, this molecule can eliminate the inflammatory microenvironment in the liver. Meanwhile, EGCG can also downregulate liver fibrosis-associated TGF-β/SMAD and PI3K/Akt/FoxO1 signaling and the expression of collagen I-α1, tissue inhibitor of metalloproteinase 1 (TIMP-1), and α-smooth muscle actin (a-SMA). In addition, the renin-angiotensin system (RAS), which is crucial for the development of liver fibrosis, is also significantly suppressed after EGCG treatment [56,57,58,59]. Hence, EGCG has a valuable role in inhibiting the development of liver fibrosis.

EGCG can also inhibit the development of NAFLD by regulating autophagy and apoptosis. EGCG is known to promote the autophagy flux and increase the conversion of LC3I to LC3II. In hepatocytes, it also increases Beclin1 expression and decreases the expression of P62, which promote autophagy. Such changes are associated with the inhibition of ROS/MAPK signaling, which also reduces the Bad/Bcl-xl ratio, inhibiting the degradation of caspase-3/9 and PARP and reducing apoptosis in hepatocytes [60]. The intestinal microbiota is a hot area of research, and studies have shown that EGCG can also modulate the metabolism of bile acids and inhibit the development of NAFLD by altering the intestinal flora [61,97]. Oxidative stress is thought to be a major cause of hepatic fat accumulation and subsequent liver injury in NAFLD. Peroxidation of plasma and cell membranes may lead to direct cellular necrosis or apoptosis and appears to be responsible for necroinflammation. Moreover, MDA and other reactive lipid derivatives have the potential to amplify intracellular damage [98,99]. EGCG significantly reduces MDA levels in the liver and increases SOD levels. Ischemia–reperfusion experiments have shown that EGCG enhances GSH levels in the liver and also improves the liver’s antioxidant capacity [100,101]. Together, this suggests that EGCG has a significant anti-NAFLD therapeutic effect and could be a potential drug for NAFLD treatment.

### 3.5. Alcohol-Related Fatty Liver Disease

AFLD, as the name implies, is caused by long-term and excessive alcohol consumption, leading to liver cell damage, disrupting their metabolism, and causing fat accumulation. In AFLD, the amount of fatty tissue in the liver exceeds 5% of the total weight of the liver. The most direct strategy for AFLD prevention is abstinence from alcohol. In addition, some drugs can also help reduce the damage caused by alcohol to liver cells and help in AFLD treatment. Studies have demonstrated a therapeutic role for EGCG in the prevention of AFLD. Oxidative stress and reduced antioxidant capacity are important factors causing alcohol-induced liver injury [102,103]. Studies have shown that EGCG can reduce alcohol-induced ROS and lipid peroxidation levels, maintain intracellular GSH levels, preserve CYP2E1-dependent oxidative regulation, and reduce the liver cell damage caused by oxidative injury [62]. EGCG could increase the expression of phospho-acetyl CoA carboxylase (p-ACC) and carnitine palmitoyl-transferase 1 (CPT-1), reducing the oxidative damage in cells [63]. Blood iron levels are associated with the development of AFLD, and iron overload is frequently observed in patients with AFLD. This is because increased levels of iron in the blood can reduce the amount of oxygen carried by red blood cells, leading to oxidative damage. EGCG can reduce the iron content in fatty livers and thereby decrease the occurrence of oxidative stress. The effect of EGCG on iron absorption in the liver and small intestine has been implicated in this effect [64]. IL-10 secreted by TLR2 and TLR3 is thought to be effective in protecting against alcohol-induced liver injury. EGCG treatment can protect Kupffer cells against alcohol-induced liver injury by promoting IL-10 secretion via the activation of STAT3 signaling, thus exerting an anti-inflammatory effect [104]. However, few studies have examined the effect of EGCG in cases of AFLD, and this warrants further studies.

### 3.6. Hepatocellular Carcinoma

**Figure 3:**
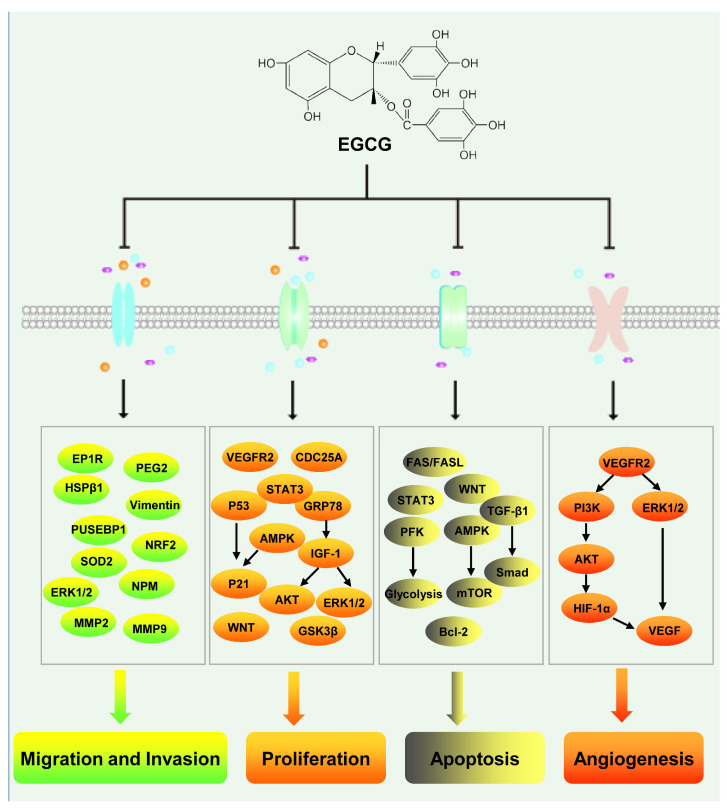
Mechanism of EGCG-induced regulation in hepatocellular carcinoma.

Hepatocellular carcinoma, a malignant tumor associated with high global mortality, ranks as the sixth most frequently diagnosed cancer worldwide. The incidence of this disease is escalating annually [105]. In the United States, between 2000 and 2016, hepatocellular carcinoma mortality increased by 43%, with a five-year survival rate of merely 18%, positioning it as the second most lethal malignancy, surpassed solely by pancreatic cancer. Alarmingly, Asian countries like China exhibit a dire five-year survival rate of only 12% [106,107]. Viral liver infections including HBV and HCV infections, alcohol abuse, and fatty liver stand as significant pathogenic factors in hepatocellular carcinoma [108]. While liver transplantation and surgical resection prove effective treatment options, their viability is hindered by factors such as donor scarcity, cirrhosis development, and late-stage diagnosis [109]. For patients with advanced hepatocellular carcinoma, sorafenib-based chemotherapy represents a potential option. Nevertheless, limited responsiveness to this type of kinase inhibitor drug is observed in numerous tumors [110]. Consequently, the quest for superior, potent, and cost-effective treatment modalities remains a pervasive focus in hepatocellular carcinoma research. One such potential contender is epigallocatechin gallate (EGCG), an abundant natural compound found in green tea and the primary component of green tea polyphenols. Notably, EGCG showcases hepatocellular carcinoma inhibition capabilities (Figure 3). 

Most tumors are difficult to cure because of the metastasis of tumor cells. EGCG has a valuable inhibitory effect on the metastasis of hepatocellular carcinoma. Matrix metalloproteinases (MMPs) play an important role in promoting tumor metastasis. In HepG2 cells, treatment with EGCG was found to inhibit invasion, likely via the EGCG-induced decrease in the secretion of MMP2 and MMP9 from HepG2 cells [111]. In SK-Hep-1 cells, EGCG was shown to inhibit the expression of MMP2 and MMP9 and suppress cell invasion and metastasis [112,113]. In one study, the treatment of HCCLM6 cells with 10 ng/mL of EGCG significantly inhibited cell migration, likely owing to the downregulation of MMP2 and MMP9 by EGCG. Meanwhile, EGCG can also downregulate the protein expression of metastasis-related upstream element (FUSE) binding protein 1 (FUBP1), heat shock protein beta 1 (HSPB1), heat shock 60-kDa protein 1 (chaperonin) (CH60), and nucleophosmin (NPM) [114]. EGCG inhibits the metastasis of hepatocellular carcinoma cells by inhibiting the thrombin-PAR1/PAR4-p42/p44 MAPK signaling axis. Low concentrations of EGCG (2 μg/mL) can inhibit the migration of HepG2 and MHCC-97H cells, and this effect may be related to the ability of EGCG to reduce the half-life of the bone bridge protein osteopontin (OPN) [115]. In the rat hepatocellular carcinoma cell line AH109A, 10 μM of EGCG was found to significantly inhibit cell invasion and adhesion via the inhibition of intracellular ROS production [116]. In addition, 100 μg/mL of EGCG could significantly inhibit the migration of HepG2 cells, likely by downregulating the expression of prostanoid EP(1) receptors and the secretion of prostaglandin 2 (PGE(2)) [117]. In addition, EGCG can inhibit the expression of miR-483-3p by inducing its methylation. Moreover, EGCG attenuates the miR-483-3p-mediated degradation of its target genes SOD2 and NRF2 and reduces intracellular ROS levels. Further, it also reverses the miR-483-3p-induced downregulation of E-cadherin and upregulation of Vimentin, inhibiting liver cell metastasis in vivo and in vitro [118,119].

Rapid proliferation and low apoptosis rates are important characteristics of tumor cells. In HepG2 hepatoma cells, treatment with EGCG was shown to significantly inhibit cell proliferation in a dose-dependent manner, with an IC50 value of 147.26 ± 5.3 μM. This outcome was primarily achieved by inhibiting the cell cycle via the activation of P53/P21 signaling and promoting apoptosis via the activation of Fas/Fasl signaling through P53 activation and Bax upregulation [120]. EGCG can also inhibit cell proliferation by activating AMPK in a P53-independent manner. In P53-positive HepG2 cells, EGCG activates AMPK and increases the expression of P21, leading to cell cycle arrest in G2 and the inhibition of cell proliferation. In contrast, in P53-mutated Hep3B cells, AMPK can inhibit cell proliferation by activating AMPK/mTOR signaling and inducing apoptosis [121]. In addition, EGCG was found to bind to STAT3 in BEL-7402 and QGY-7703 cells, inhibiting STAT3 phosphorylation and altering the expression of target genes downstream to STAT3, inhibiting cell growth and promoting apoptosis. EGCG is also known to inhibit the activation of VEGFR2-mediated signaling by downregulating the protein levels of VEGFR2, thereby inhibiting hepatoma growth [122]. In a rat hepatocellular carcinoma model, EGCG inhibited the growth of hepatocellular carcinoma by upregulating P21waf1/Cip and downregulating CDC25A expression [123]. EGCG can also attenuate the growth of hepatocellular carcinoma cells by inhibiting the IGF-1/IGF-1R axis and ERK, AKT, STAT3, and GSK3β signals [124]. This effect of EGCG may be mediated by glucose-regulated protein 78 (GRP78), as one study showed that EGCG can inhibit hepatocellular carcinoma by specifically inhibiting the expression of GRP78—a molecule upstream of IGF-1 that can downregulate IGF-1-induced activation of PI3K and MAPK signaling [125]. The combination of EGCG and theaflavin (TF) has also been shown to effectively downregulate Wnt signaling in hepatocellular carcinoma cells, inhibit cell proliferation, and promote apoptosis, thereby inhibiting the development of hepatocellular carcinoma [126]. EGCG can also effectively inhibit glycolysis and thus promote cell apoptosis in hepatocellular carcinoma cells with high aerobic activity by inhibiting the activity of phosphofructokinase (PFK), the rate-limiting enzyme of glycolysis [127,128]. In HepG2 hepatocellular carcinoma cells, EGCG can also induce apoptosis by activating TGF-β1/Smad signaling and promoting a G1/S phase cell cycle block [129]. In SMMC7721 cells, EGCG can induce apoptosis by inhibiting AKT signaling and causing an S-phase arrest [130]. Further, EGCG can also exert effects on microRNAs. Studies have shown that EGCG can upregulate the expression of miR-16, thereby reducing the expression of the miR-16 target gene BCL-2 and promoting apoptosis in hepatocellular carcinoma cells [131]. Studies are now increasingly reporting that EGCG can inhibit the proliferation of tumor cells and promote their apoptosis. Therefore, the anti-tumor effects of EGCG are expected to become more widely accepted.

Angiogenesis is essential for the growth of solid tumors, and tumor diameters cannot exceed 2 mm without angiogenesis [132]. EGCG also shows valuable effects on angiogenesis in hepatocellular carcinoma. In hepatocellular carcinoma, EGCG inhibits the expression of HIF-1α and VEGF, two molecules with important roles in tumor angiogenesis [132,133]. In mice fed with EGCG, the growth of transplanted tumors is significantly inhibited. This may be because EGCG inhibits VEGFR2 signaling in tumors and downregulates the PI3K and ERK signaling pathways, leading to the downregulation of VEGF mRNA. VEGF is an important promoter of angiogenesis; therefore, the EGCG-induced inhibition of hepatocellular carcinoma growth likely results from the inhibition of angiogenesis in tumor tissues [122]. In vivo and in vitro experiments have confirmed that EGCG and its derivatives can inhibit angiogenesis in hepatocellular carcinoma. Treatment with EGCG or the EGCG derivative Y6 was found to significantly inhibit tube formation in hepatocellular carcinoma cells and angiogenesis in transplanted tumors, likely via the downregulation of the MAPK/ERK1/2 and PI3K/AKT/HIF-1α/VEGF pathways. Methylated EGCG could also significantly inhibit angiogenesis in liver graft tumors and suppress the growth of graft tumors. The downregulation of VEGFR2 and P42/P44 MAPK signaling by methylated EGCG, which inhibits the growth of hepatocellular carcinoma cells and the formation of blood vessels in the tumor microenvironment, has been implicated in this effect [134]. Although there are few studies reporting that EGCG can inhibit angiogenesis in hepatocellular carcinoma, more studies and promising results are expected in the future.

In summary, EGCG has demonstrated an effective inhibition of hepatocellular carcinoma cell development, including proliferation inhibition and promotion of apoptosis. Moreover, it can hinder angiogenesis and reduce the tumor’s nutrient supply. Additionally, EGCG inhibits metastasis by downregulating the expression of MMP2, MMP9, and OPN, while also participating in epigenetic modifications. Although the molecular mechanism by which EGCG inhibits hepatocellular carcinoma is not well understood and needs to be explored further, EGCG appears to show good potential as a therapeutic agent for hepatocellular carcinoma.

## 4. Applications of EGCG and Its Potential in the Fight Against Liver Diseases

Green tea has a long and rich history and is well-recognized as a healthy beverage worldwide. Green tea is rich in tea polyphenols, which have valuable health-promoting effects. EGCG is the most abundant polyphenolic compound in GTE and has anti-inflammatory, antioxidant, antibacterial, antiviral, anti-cardiovascular disease, and anti-cancer effects [27]. Many people consume EGCG in their daily diet. In the United States, GTE is sometimes labeled as a natural flavor with antioxidant activity and is used in foods such as oils (U.S. FDA, 2012). In the EU, GTE meets the definition of natural flavoring substances as stated in Council Directive 88/388/EEC, and it can be used in all foods except those for which the use of flavorings is restricted. Because of its antioxidant and antibacterial properties, EGCG is used to extend the shelf life of food [135]. EGCG is also used in cosmetics owing to its excellent antioxidant effects and biosafety. EGCG-based health products that eliminate free radicals from the body and improve bodily functions have also appeared on the market. The safety of EGCG as a dietary supplement is also of great concern. Toxicity tests have confirmed that daily supplementation with a suitable dose of EGCG is well-tolerated by the body, and up to 600 mg of EGCG per day can be consumed without any adverse effects on human liver function [136,137]. In addition, EGCG has also been found to have good therapeutic effects in the prevention and treatment of liver-related diseases. HCV infection is an important contributor to viral hepatitis, and in a treatment regimen for genotype 4 HCV infection, the HCV viral load was found to decrease more rapidly in patients treated with EGCG. This could be because EGCG interferes with viral entry, thus improving treatment efficacy and preventing relapse [138]. In a similar clinical trial, EGCG showed a significant anti-HCV effect [139]. This suggests that EGCG is valuable in the prevention and treatment of HCV infection and can be considered a candidate agent for the same.

EGCG has shown great potential in the treatment of hepatocellular carcinoma (Table 2). In doxorubicin-resistant hepatocellular carcinoma cells (BEL-7404), the addition of a high concentration of EGCG alone was found to have no significant effect on cell proliferation. However, when a combination of doxorubicin and low-concentration EGCG was administered, the growth of BEL-7404 cells and transplanted BEL-7404 tumors was more significantly inhibited than in the doxorubicin group alone. Therefore, EGCG can improve the killing effect of doxorubicin in tumor cells and enhance the sensitivity of doxorubicin-resistant cells. EGCG may reverse multidrug resistance mechanisms in hepatocellular carcinoma cells; it may inhibit the expression of the multiple drug resistance 1 (MDR1) gene or downregulate P-glycoprotein (P-gp), reducing P-glycoprotein-mediated drug pumping activity and causing intracellular doxorubicin accumulation [140]. Autophagy is commonly observed in cells resistant to multiple chemotherapeutic agents. EGCG primarily promotes the killing activity of doxorubicin against hepatocellular carcinoma by inhibiting autophagy in tumor cells and reducing the resistance of tumor cells to doxorubicin [141]. 5-fluorouracil (5-FU), which is also an inhibitor of DNA synthesis, is widely used in the clinical treatment of tumors. However, prolonged treatment with 5-FU can lead to tumor resistance [142]. The combination of EGCG and 5-FU significantly inhibits the growth of Hep3B hepatocellular carcinoma cells, even when the concentration of EGCG is very low (5 μM). This effect is observed because EGCG, in synergy with 5-FU, activates AMPK and reduces COX2 expression and PEG2 secretion while inhibiting AKT signaling and suppressing the growth of tumor cells [143]. These results suggest that EGCG can be used as an adjunctive agent to improve the efficacy of anti-tumor drugs against hepatocellular carcinoma. In recent years, studies have found that metformin, a commonly used hypoglycemic drug, has anti-tumor effects. EGCG combined with metformin can significantly inhibit the growth of hepatocellular carcinoma. This combination provides a greater reduction in the expression of cyclinD1, survivin, and VEGF in hepatocellular carcinoma than either agent alone, while enhancing the expression of the pro-apoptotic gene caspase3. EGCG combined with metformin is believed to inhibit cell proliferation by inducing cell cycle arrest in hepatocellular carcinoma cells. This combination also inhibits angiogenesis and promotes apoptosis in hepatocellular carcinoma cells, ultimately inhibiting the growth of hepatocellular carcinoma [144]. Prostaglandin receptor (EP1) can promote tumor invasion and metastasis. Compared with EGCG alone, the combination of EGCG and an EP1 inhibitor can significantly inhibit the survival and migration of hepatocellular carcinoma cells [145]. The tumor necrosis factor-related apoptosis-inducing ligand (TRAIL) is a pro-apoptotic cytokine belonging to the TNF family. It can induce apoptosis by binding to death receptors (DRs) on the cell surface. TRAILR1/DR4 and TRAILR2/DR5 signaling promote apoptosis, but some hepatocellular carcinoma cells are resistant to TRAIL-induced apoptosis. In HepG2 cells, the combined use of TRAIL and EGCG was found to significantly enhance the sensitivity of cells to TRAIL and thereby induce apoptosis. The underlying mechanism may involve the enhancement of caspase3 activity in cells, induction of DR4 and DR5 expression, and downregulation of the apoptosis suppressor Bcl-2, ultimately leading to apoptosis [146]. Although EGCG is not currently used as an adjuvant drug in the treatment of liver-related diseases, these preclinical and clinical studies have demonstrated that it can play a significant role in the treatment of liver-related diseases and could be a potential drug candidate.

## 5. Discussion and Perspectives

EGCG, as the core active component of green tea polyphenols, exhibits a multi-faceted regulatory potential in liver protection mechanisms. As the most vital metabolic organ in the human body, the liver is constantly exposed to exogenous factors (including dietary toxins, drug metabolism, and viral infections) and endogenous factors (such as autoimmune hepatitis) that contribute to liver damage. Through targeted modulation of key signaling networks, EGCG demonstrates significant therapeutic benefits in various liver diseases. In the context of viral hepatitis, EGCG inhibits host cell invasion and viral replication of HBV/HCV, reducing viral load and effectively reducing the pro-inflammatory response induced by HCV infection. In the case of autoimmune hepatitis, EGCG regulates the hepatic inflammatory microenvironment by inhibiting the NF-κB/STAT3 signaling axis and promotes a balanced state of autophagy and apoptosis to enhance the hepatocyte’s resistance to damage. For metabolism-related fatty liver disease, EGCG effectively retards the progression of liver fibrosis by intervening in glucose metabolic reprogramming, lipid homeostasis imbalance, and bile acid toxicity through multi-target intervention. In the field of liver cancer treatment, EGCG exhibits a triple effect, inhibiting tumor proliferation, invasion, and angiogenesis by downregulating the MAPK/AKT/STAT3 signaling cascade. It further achieves synergistic regulation by altering the P53/P21-AMPK metabolic axis and BCL-2/VEGFA protein network. Furthermore, the combined use with chemotherapy drugs highlights the clinical translational value of EGCG.

Despite the advantageous multi-target regulatory properties of EGCG, its clinical application is hindered by challenges such as low bioavailability, short metabolic half-life, and inadequate liver targeting. To overcome these limitations, we propose systematic solutions. At the mechanistic level, integrating single-cell spatial transcriptome and epigenetic techniques can provide dynamic insights into the spatio-temporal regulation of key pathways, such as Nrf2/ARE and SIRT1/PGC-1α. Additionally, clarifying the enterohepatic dialogue mechanism through the intestinal microbiota–bile acid axis with the aid of organoid-organ chip systems is suggested. In terms of delivery system innovation, a GalNAc-modified ROS-responsive nanocrystalline prodrug system can improve the accumulation efficiency of liver parenchymal cells using metal chelation technology, while the development of a long-acting sustained-release formulation with PLGA microspheres aims to overcome pharmacokinetic limitations. Furthermore, structural optimization options include constructing mitochondria-targeted EGCG-TPP conjugates to enhance antioxidant stress efficacy or designing EGCG-sorafenib hybrid molecules to achieve synergistic anti-angiogenic effects. In the context of clinical translation, the establishment of a personalized drug delivery system based on population pharmacokinetic modeling and the use of 3D bioprinting liver chip platforms for toxicity threshold assessment would be valuable.

The pleiotropic effects of EGCG are noteworthy and may be attributed to its unique “molecular glue” property, which dynamically regulates multiple signaling proteins through interactions with their hydrophobic domains. While this multi-target property provides therapeutic advantages, it also contributes to the complexity of the underlying mechanism of action. To address this complexity, future studies should establish a “computation-experiment” closed-loop validation system. This entails using deep learning algorithms to predict EGCG–protein interaction networks, validating key targets through CRISPR interference screening, and implementing a multi-omics dynamic monitoring platform (phosphorylation proteome/metabolome/epigenome) to analyze dose–effect relationships. Only by comprehensively investigating the mechanism and conducting innovation in dosage forms, along with clinical validation, can EGCG transition from a multifaceted molecule in the laboratory to a precise therapeutic agent for liver diseases in clinical practice. Such efforts will provide innovative solutions for the prevention, treatment, and rehabilitation of liver diseases in a three-level control system.

## Figures and Tables

**Table 1:** The therapeutic roles of EGCG in viral hepatitis, autoimmune hepatitis, fatty liver, and hepatocellular carcinoma.

No.	Disease Type	Model/Species	Test Compound	Dose	Administration Method	Effects	References
1	Hepatitis B virus	HepG2-N10 cells	GTE (contains EGCG)	-	-	The 50% effective concentrations of GTE on HBsAg, HBeAg, extracellular HBV DNA, and intracellular HBV DNA were 5.02, 5.681, 19.81, and 10.76 mu g/mL, respectively.	[36]
2	Hepatitis B virus	HuS-E/2 and Huh7 cells	EGCG	50 μM	-	EGCG has the potential to inhibit HBV entry into host cells, with an inhibition rate of up to 80% at a concentration of 50 μM; EGCG induced clathrin-dependent endocytosis of NTCP from the plasma membrane followed by protein degradation; EGCG inhibited the clathrin-mediated endocytosis of transferrin.	[37]
3	Hepatitis B virus	HuS-E/2 cells	EGCG	0, 10, and 20 μM	-	EGCG treatment during infection led to a dose-dependent reduction in HBV rcDNA and HBsAg mRNA in HuS-E/2 cells, with HBV mRNA levels being decreased by 80% compared to control cells when treated with 10 μM of EGCG. The half-maximal inhibitory concentration was estimated to be below 10 μM.	[38]
4	Hepatitis B virus	Hu-FRG mice	EGCG	50 mg/kg	Injected intraperitoneally	EGCG inhibited HBV infection, the expression of FAH and HBcAg.	[38]
5	Hepatitis B virus	HepG2.117 cells	EGCG	0, 50, 100, 200, and 400 μM	-	EGCG inhibited HBV replication by disrupting the synthesis of HBV replicative intermediates, leading to a decrease in the production of HBV covalently closed circular DNA.	[39]
6	Hepatitis B virus	Hep3B2.1-7 cells	EGCG	100 μM	-	EGCG could have strong effects on HBsAg and HBeAg levels and prevent HBV DNA replication.	[40]
7	Hepatitis B virus	HepG2 2.2.15 cells	EGCG	0.11–0.44 μM	-	EGCG effectively suppressed the secretion of HBsAg and HBeAg in a dose- and time-dependent manner.	[41]
8	Hepatitis B virus	HepG2.2.15 cells	EGCG	12.5–50 μM	-	EGCG dose-dependently inhibited HBV gene expression and replication; EGCG significantly activated ERK1/2 MAPK signaling, and slightly activated p38 MAPK and JAK2/STAT3 signaling	[42]
9	Hepatitis B virus	HBV infection mice	EGCG	25 mg/kg	Injected intraperitoneally	EGCG inhibited HBV gene expression and replication, which involves ERK1/2-mediated downregulation of HNF4α.	[42]
10	Hepatitis B virus	HepG2-N10 cells	EGCG	0–100 μM	-	EGCG inhibited the regulation of HBV antigens by interacting with FXRα, which in turn regulates HBV antigens and activates Enhll core promoter activity through the FXRα/RXRα axis.	[43]
11	Hepatitis C virus	Huh7 HCVcc cells	EGCG	10 μg/mL	-	EGCG enhanced miR-548m expression and repressing CD81 receptor to reduce cellular infectivity.	[44]
12	Hepatitis C virus	Huh-7 cells	EGCG	50 μM	-	EGCG altered the viral particle structure and impaired its attachment to the cell surface.	[45]
13	Hepatitis C virus	Huh-7.5 cells and Primary human hepatocytes	EGCG	0–100 μM	-	EGCG inhibited cell-culture-derived HCV entry into hepatoma cell lines as well as primary human hepatocytes.	[46]
14	Hepatitis C virus	Huh-7 cells	EGCG	0–10 μM	-	EGCG significantly enhanced HCV dsRNAs-induced expression of IFN-lambda 1, TLR3, RIG-I, and antiviral ISGs in hepatocytes.	[47]
15	Autoimmune hepatitis	Bovine hepatocytes	EGCG	50 μM	-	GCG significantly attenuates inflammatory reactions and oxidative stress under the control of the NF-κB and MAPK cascades and the Nrf2 complex.	[48]
16	Autoimmune hepatitis	Balb/C mice received intraperitoneal injection with GalN (700 mg/kg) and LPS (10 μg/kg)	EGCG	10, 25, and 50 mg/kg	Oral gavage	EGCG was hepatoprotective via inhibition of MAPK/NF-κB signaling and activation of the Nrf2 cascade.	[48]
17	Autoimmune hepatitis	Balb/C mice were injected with ConA (25 mg/kg)	EGCG	10 and 30 mg/kg	Oral gavage	EGCG attenuated liver injury in ConA-induced hepatitis by downregulating IL-6/JAKs/STAT3/BNIP3-mediated apoptosis and autophagy.	[49]
18	Non-alcoholic fatty liver disease	HFD-fed mice	EGCG	10, 20, and 40 mg/kg	Injected intraperitoneally	EGCG demonstrated dose-dependent improvement in hepatic morphology and function, reduction in body weight, and alleviation of hyperlipidemia, hyperglycemia, hyperinsulinemia, and insulin resistance in NAFLD mice. Additionally, EGCG dose-dependently enhanced insulin clearance and upregulated IDE protein expression and enzyme activity in the liver of NAFLD mice.	[50]
19	Non-alcoholic fatty liver disease	HepG2 cells	EGCG	10 μM	-	EGCG was capable of enhancing insulin-mediated glucose and lipid metabolism by regulating enzymes involved in glycogen synthesis and lipogenesis.	[51]
20	Non-alcoholic fatty liver disease	HepG2 cells	EGCG	50 μM		EGCG reduced cellular lipid accumulation in FFA-induced HepG2 cells through the activation of AMP-activated protein kinase resulting from the generation of reactive oxygen species	[52]
21	Non-alcoholic fatty liver disease	HFD-fed mice	GTE (contain EGCG)	50 mg/kg	Oral gavage	The effects of decaffeinated green tea extract may be related to the activation of AMPK via LKB1 in the liver of HFD-fed mice.	[53]
22	Non-alcoholic fatty liver disease	HFD-C57BL/6 mice	GTE (contain EGCG)	1% (*w*/*w*)	Additive feed	EGCG decreased post-prandial triglyceride and glycogen content in liver, increased oxidation of dietary lipids, and decreased incorporation of dietary 13C-enriched lipids into fat tissues, liver, and skeletal muscle. EGCG dose-dependently reversed high-fat diet-induced effects on intestinal substrate transporters (CD36, FATP4, and SGLT1) and downregulated lipogenesis-related genes (ACC, FAS, and SCD1) in the liver.	[54]
23	Non-alcoholic fatty liver disease	HFD-fed mice	EGCG	50 mg/kg	Oral gavage	EGCG also increases the oxidation of long-chain fatty acids by increasing the activity of the mitochondrial complex, thus halting NAFLD progression.	[55]
24	Non-alcoholic steatohepatitis	MCD diet mice	EGCG	25, 50, and 100 mg/kg	Oral gavage	EGCG attenuated NASH induced by MCD diet associated with ameliorating fibrosis, oxidative stress, and hepatic inflammation.	[56]
25	Non-alcoholic fatty liver disease	HFD-fed rats	EGCG	50 mg/kg	Injected intraperitoneally	EGCG reduced the severity of liver injury in an experimental model of NAFLD associated with lower concentration of pro-fibrogenic, oxidative stress, and pro-inflammatory mediators partly through modulating the activities of the TGF/SMAD, PI3 K/Akt/FoxO1, and NF-kappa B pathways.	[57]
26	Non-alcoholic steatohepatitis	CCL4-induced rats	EGCG	0.1% (*w*/*w*)	Injected intraperitoneally	EGCG inhibited the development of hepatic premalignant lesions by improving liver fibrosis, inhibiting RAS activation, and attenuating inflammation and oxidative stress.	[58]
27	Non-alcoholic fatty liver disease	HFD-induced mice	EGCG	25 and 50 mg/kg	Oral gavage	EGCG impacted M1/M2 macrophage polarization.	[59]
28	Non-alcoholic fatty liver disease	HFD-induced mice	EGCG	50 mg/kg	Oral gavage	EGCG alleviated HFD-induced NAFLD possibly by decreasing apoptosis and increasing autophagy via the ROS/MAPK pathway.	[60]
29	Non-alcoholic fatty liver disease	HFD-induced mice	EGCG	0.32% (*w*/*w*)	Additive feed	EGCG could alter bile acid metabolism, especially taurine deconjugation, and suppress fatty liver disease by improving the intestinal luminal environment.	[61]
30	Alcohol-related fatty liver disease	Alcohol-fed rats	EGCG	200 mg/kg	Oral gavage	EGCG inhibited fatty acid synthesis and the alleviation of lipid peroxidation through the downregulation of the mRNA and protein expression of TNF-alpha, SREBP1c, and CYP2E1 and the upregulation of the mRNA and protein expression of ADH1, ALDH2, Lipin-1, PPAR α, AMPK, and PGC-1 α, thereby promoting the oxidative decomposition of fatty acids and reducing the synthesis of cholesterol and glucose.	[62]
31	Alcohol-related fatty liver disease	Alcohol-fed rats	EGCG	3 g/L	Additive feed	EGCG markedly reversed the effect of ethanol on hepatic p-ACC and CPT-1 levels, prevented ethanol-induced hepatotoxicity, and inhibits the development of a fatty liver.	[63]
32	Alcohol-related fatty liver disease	Alcohol-fed mice	EGCG	10, 20, and 30 mg/kg	Injected intraperitoneally	EGCG ameliorated liver injuries; decreased serum iron level, hepatic iron levels, and liver MDA contents; increased hepcidin mRNA level; and decreased Tf and TfR1 protein expression in the liver.	[64]

**Table 2:** Summary of the application of EGCG incorporation with anti-tumor compounds in HCC.

No.	Compounds	Effects on HCC	Proposed Mechanisms of Anti-Hepatocarcinogenesis	References
1	EGCG + doxorubicin	Inhibit proliferation, enhance cell sensitivity to doxorubicin	Downregulate expression of MDR1 and p-glycoprotein, inhibit autophagy	[141]
2	EGCG + 5-FU	Inhibit proliferation	Activate AMPK, decrease COX2 expression and reduce PEG2 secretion, inhibit AKT signaling	[143]
3	EGCG + Metformin	Cell cycle arrest, promote apoptosis, inhibit angiogenesis	Downregulate expression of cyclinD1, survivin and VEGFA, upregulate caspase3	[144]
4	EGCG + EP1 inhibitor	Inhibit migration and survival	Suppress EP1 receptor expression and PGE2 production	[145]
5	EGCG + TRAIL	Enhanced cell sensitivity to TRAIL, promote apoptosis	Enhance caspase3 activity, induce DR4/DR5 expression, downregulate Bcl-2 expression	[146]

## Abbreviations

EGCG: Epigallocatechin gallate; HBV: Hepatitis B virus; HCV: Hepatitis C virus; EC: Epicatechin; EGC: Epigallocatechin; ECG: Epicatechin gallate; Ag: antigen; ROS: reactive oxygen species; NTCP: sodium-taurocholate co-transporting polypeptide; HNF4α: hepatocyte nuclear factor 4α; TLR3: Toll-like receptor 3; ISGs: IFN-stimulated genes; ConA: concanavalin; ALT: alanine aminotransferase; MDA: malondialdehyde; GSH: glutathione; SOD: superoxide dismutase; TG: triglycerides; NAFLD: non-alcoholic fatty liver disease; AFLD: alcoholic-related fatty liver disease; GSK: glycogen synthase kinase; LDL-R: low-density lipoprotein receptor; TLR2: Toll-like receptor 2; GRP78: glucose-regulated protein 78; PFK: phosphofructokinase; MDR1: multiple drug resistance 1; P-gp: P-glycoprotein; TRAIL: tumor necrosis factor-related apoptosis-inducing ligand.

## References

[1] Lanini S., Ustianowski A., Pisapia R., Zumla A., Ippolito G. (2019). Viral Hepatitis: Etiology, Epidemiology, Transmission, Diagnostics, Treatment, and Prevention. Infect. Dis. Clin. N. Am..

[2] Cotter T.G., Rinella M. (2020). Nonalcoholic Fatty Liver Disease 2020: The State of the Disease. Gastroenterology.

[3] Collaborators G.B.D.O., Afshin A., Forouzanfar M.H., Reitsma M.B., Sur P., Estep K., Lee A., Marczak L., Mokdad A.H., Moradi-Lakeh M. (2017). Health Effects of Overweight and Obesity in 195 Countries over 25 Years. N. Engl. J. Med..

[4] Heneghan M.A., Yeoman A.D., Verma S., Smith A.D., Longhi M.S. (2013). Autoimmune hepatitis. Lancet.

[5] Kulik L., El-Serag H.B. (2019). Epidemiology and Management of Hepatocellular Carcinoma. Gastroenterology.

[6] Brody H. (2019). Tea. Nature.

[7] Drew L. (2019). The growth of tea. Nature.

[8] Zhou J., Ho C.T., Long P., Meng Q., Zhang L., Wan X. (2019). Preventive Efficiency of Green Tea and Its Components on Nonalcoholic Fatty Liver Disease. J. Agric. Food Chem..

[9] Li C.F., Zhu Y., Yu Y., Zhao Q.Y., Wang S.J., Wang X.C., Yao M.Z., Luo D., Li X., Chen L. (2015). Global transcriptome and gene regulation network for secondary metabolite biosynthesis of tea plant (*Camellia sinensis*). BMC Genom..

[10] Li P., Liu A., Xiong W., Lin H., Xiao W., Huang J., Zhang S., Liu Z. (2020). Catechins enhance skeletal muscle performance. Crit. Rev. Food Sci. Nutr..

[11] James A., Wang K., Wang Y. (2023). Therapeutic Activity of Green Tea Epigallocatechin-3-Gallate on Metabolic Diseases and Non-Alcoholic Fatty Liver Diseases: The Current Updates. Nutrients.

[12] Alam M., Gulzar M., Akhtar M.S., Rashid S., Zulfareen, Tanuja, Shamsi A., Hassan M.I. (2024). Epigallocatechin-3-gallate therapeutic potential in human diseases: Molecular mechanisms and clinical studies. Mol. Biomed..

[13] Mokra D., Joskova M., Mokry J. (2023). Therapeutic Effects of Green Tea Polyphenol (−)-Epigallocatechin-3-Gallate (EGCG) in Relation to Molecular Pathways Controlling Inflammation, Oxidative Stress, and Apoptosis. Int. J. Mol. Sci..

[14] Qu Z., Liu A., Li P., Liu C., Xiao W., Huang J., Liu Z., Zhang S. (2021). Advances in physiological functions and mechanisms of (−)-epicatechin. Crit. Rev. Food Sci. Nutr..

[15] Bakun P., Mlynarczyk D.T., Koczorowski T., Cerbin-Koczorowska M., Piwowarczyk L., Kolasinski E., Stawny M., Kuzminska J., Jelinska A., Goslinski T. (2023). Tea-break with epigallocatechin gallate derivatives—Powerful polyphenols of great potential for medicine. Eur. J. Med. Chem..

[16] Zhang S., Mao B., Shumao C., Zhang Q., Zhao J., Tang X., Chen W. (2024). Absorption, metabolism, bioactivity, and biotransformation of epigallocatechin gallate. Crit. Rev. Food Sci. Nutr..

[17] Chow H.H.S., Cai Y., Hakim I.A., Crowell J.A., Shahi F., Brooks C.A., Dorr R.T., Hara Y., Alberts D.S. (2003). Pharmacokinetics and safety of green tea polyphenols after multiple-dose administration of epigallocatechin gallate and polyphenon E in healthy individuals. Clin. Cancer Res. Off. J. Am. Assoc. Cancer Res..

[18] Misaka S., Kawabe K., Onoue S., Werba J.P., Giroli M., Kimura J., Watanabe H., Yamada S. (2013). Development of rapid and simultaneous quantitative method for green tea catechins on the bioanalytical study using UPLC/ESI-MS. Biomed. Chromatogr..

[19] Zhu M., Chen Y., Li R.C. (2000). Oral absorption and bioavailability of tea catechins. Planta Medica.

[20] Lin L.-C., Wang M.-N., Tseng T.-Y., Sung J.-S., Tsai T.-H. (2007). Pharmacokinetics of (−)-epigallocatechin-3-gallate in conscious and freely moving rats and its brain regional distribution. J. Agric. Food Chem..

[21] Nakagawa K., Miyazawa T. (1997). Absorption and distribution of tea catechin, (−)-epigallocatechin-3-gallate, in the rat. J. Nutr. Sci. Vitaminol..

[22] Zagury Y., Kazir M., Livney Y.D. (2019). Improved antioxidant activity, bioaccessibility and bioavailability of EGCG by delivery in β-lactoglobulin particles. J. Funct. Foods.

[23] Yang C.S., Sang S., Lambert J.D., Lee M.-J. (2008). Bioavailability issues in studying the health effects of plant polyphenolic compounds. Mol. Nutr. Food Res..

[24] Sun W., Yang Y., Wang C., Liu M., Wang J., Qiao S., Jiang P., Sun C., Jiang S. (2024). Epigallocatechin-3-gallate at the nanoscale: A new strategy for cancer treatment. Pharm. Biol..

[25] Liu J.B., Li J.L., Zhuang K., Liu H., Wang X., Xiao Q.H., Li X.D., Zhou R.H., Zhou L., Ma T.C. (2018). Epigallocatechin-3-gallate local pre-exposure application prevents SHIV rectal infection of macaques. Mucosal Immunol..

[26] Isbrucker R.A., Edwards J.A., Wolz E., Davidovich A., Bausch J. (2006). Safety studies on epigallocatechin gallate (EGCG) preparations. Part 2: Dermal, acute and short-term toxicity studies. Food Chem. Toxicol. Int. J. Publ. Br. Ind. Biol. Res. Assoc..

[27] Yates A.A., Erdman J.W., Shao A., Dolan L.C., Griffiths J.C. (2017). Bioactive nutrients—Time for tolerable upper intake levels to address safety. Regul. Toxicol. Pharmacol..

[28] Galati G., Lin A., Sultan A.M., O’Brien P.J. (2006). Cellular and in vivo hepatotoxicity caused by green tea phenolic acids and catechins. Free. Radic. Biol. Med..

[29] Samavat H., Newman A.R., Wang R., Yuan J.M., Wu A.H., Kurzer M.S. (2016). Effects of green tea catechin extract on serum lipids in postmenopausal women: A randomized, placebo-controlled clinical trial. Am. J. Clin. Nutr..

[30] Hu J., Webster D., Cao J., Shao A. (2018). The safety of green tea and green tea extract consumption in adults—Results of a systematic review. Regul. Toxicol. Pharmacol..

[31] Wang L., Li P., Feng K. (2023). EGCG adjuvant chemotherapy: Current status and future perspectives. Eur. J. Med. Chem..

[32] Wang Y.-Q., Lu J.-L., Liang Y.-R., Li Q.-S. (2018). Suppressive Effects of EGCG on Cervical Cancer. Molecules.

[33] Lambert J.D., Kennett M.J., Sang S., Reuhl K.R., Ju J., Yang C.S. (2010). Hepatotoxicity of high oral dose (−)-epigallocatechin-3-gallate in mice. Food Chem. Toxicol..

[34] Younes M., Aggett P., Aguilar F., Crebelli R., Dusemund B., Filipic M., Frutos M.J., Galtier P., Gott D., Gundert-Remy U. (2018). Scientific opinion on the safety of green tea catechins. EFSA J..

[35] Sanlier N., Gokcen B.B., Altug M. (2018). Tea consumption and disease correlations. Trends Food Sci. Technol..

[36] Xu J., Wang J., Deng F., Hu Z., Wang H. (2008). Green tea extract and its major component epigallocatechin gallate inhibits hepatitis B virus in vitro. Antivir. Res..

[37] Huang H.C., Tao M.H., Hung T.M., Chen J.C., Lin Z.J., Huang C. (2014). (−)-Epigallocatechin-3-gallate inhibits entry of hepatitis B virus into hepatocytes. Antivir. Res..

[38] Lai Y.H., Sun C.P., Huang H.C., Chen J.C., Liu H.K., Huang C. (2018). Epigallocatechin gallate inhibits hepatitis B virus infection in human liver chimeric mice. BMC Complement. Altern. Med..

[39] He W., Li L.X., Liao Q.J., Liu C.L., Chen X.L. (2011). Epigallocatechin gallate inhibits HBV DNA synthesis in a viral replication—Inducible cell line. World J. Gastroenterol..

[40] Karamese M., Aydogdu S., Karamese S.A., Altoparlak U., Gundogdu C. (2015). Preventive effects of a major component of green tea, epigallocathechin-3-gallate, on hepatitis-B virus DNA replication. Asian Pac. J. Cancer Prev..

[41] Pang J.Y., Zhao K.J., Wang J.B., Ma Z.J., Xiao X.H. (2014). Green tea polyphenol, epigallocatechin-3-gallate, possesses the antiviral activity necessary to fight against the hepatitis B virus replication in vitro. J. Zhejiang Univ. Sci. B.

[42] Wang Z.Y., Li Y.Q., Guo Z.W., Zhou X.H., Lu M.D., Xue T.C., Gao B. (2020). ERK1/2-HNF4alpha axis is involved in epigallocatechin-3-gallate inhibition of HBV replication. Acta Pharmacol. Sin..

[43] Xu J., Gu W., Li C., Li X., Xing G., Li Y., Song Y., Zheng W. (2016). Epigallocatechin gallate inhibits hepatitis B virus via farnesoid X receptor alpha. J. Nat. Med..

[44] Mekky R.Y., El-Ekiaby N., El Sobky S.A., Elemam N.M., Youness R.A., El-Sayed M., Hamza M.T., Esmat G., Abdelaziz A.I. (2019). Epigallocatechin gallate (EGCG) and miR-548m reduce HCV entry through repression of CD81 receptor in HCV cell models. Arch. Virol..

[45] Calland N., Sahuc M.E., Belouzard S., Pene V., Bonnafous P., Mesalam A.A., Deloison G., Descamps V., Sahpaz S., Wychowski C. (2015). Polyphenols Inhibit Hepatitis C Virus Entry by a New Mechanism of Action. J. Virol..

[46] Ciesek S., von Hahn T., Colpitts C.C., Schang L.M., Friesland M., Steinmann J., Manns M.P., Ott M., Wedemeyer H., Meuleman P. (2011). The green tea polyphenol, epigallocatechin-3-gallate, inhibits hepatitis C virus entry. Hepatology.

[47] Wang Y., Li J., Wang X., Pena J.C., Li K., Zhang T., Ho W. (2016). (−)-Epigallocatechin-3-Gallate Enhances Hepatitis C Virus Double-Stranded RNA Intermediates-Triggered Innate Immune Responses in Hepatocytes. Sci. Rep..

[48] Xu T., Liu R., Zhu H., Zhou Y., Pei T., Yang Z. (2022). The Inhibition of LPS-Induced Oxidative Stress and Inflammatory Responses Is Associated with the Protective Effect of (−)-Epigallocatechin-3-Gallate on Bovine Hepatocytes and Murine Liver. Antioxidants.

[49] Li S., Xia Y., Chen K., Li J., Liu T., Wang F., Lu J., Zhou Y., Guo C. (2016). Epigallocatechin-3-gallate attenuates apoptosis and autophagy in concanavalin A-induced hepatitis by inhibiting BNIP3. Drug Des. Dev. Ther..

[50] Gan L., Meng Z.J., Xiong R.B., Guo J.Q., Lu X.C., Zheng Z.W., Deng Y.P., Luo B.D., Zou F., Li H. (2015). Green tea polyphenol epigallocatechin-3-gallate ameliorates insulin resistance in non-alcoholic fatty liver disease mice. Acta Pharmacol. Sin..

[51] Kim J.J., Tan Y., Xiao L., Sun Y.L., Qu X. (2013). Green tea polyphenol epigallocatechin-3-gallate enhance glycogen synthesis and inhibit lipogenesis in hepatocytes. Biomed. Res. Int..

[52] Liu Z., Li Q., Huang J., Liang Q., Yan Y., Lin H., Xiao W., Lin Y., Zhang S., Tan B. (2013). Proteomic analysis of the inhibitory effect of epigallocatechin gallate on lipid accumulation in human HepG2 cells. Proteome Sci..

[53] Santamarina A.B., Oliveira J.L., Silva F.P., Carnier J., Mennitti L.V., Santana A.A., de Souza G.H., Ribeiro E.B., Oller do Nascimento C.M., Lira F.S. (2015). Green Tea Extract Rich in Epigallocatechin-3-Gallate Prevents Fatty Liver by AMPK Activation via LKB1 in Mice Fed a High-Fat Diet. PLoS ONE.

[54] Friedrich M., Petzke K.J., Raederstorff D., Wolfram S., Klaus S. (2012). Acute effects of epigallocatechin gallate from green tea on oxidation and tissue incorporation of dietary lipids in mice fed a high-fat diet. Int. J. Obes..

[55] Santamarina A.B., Carvalho-Silva M., Gomes L.M., Okuda M.H., Santana A.A., Streck E.L., Seelaender M., do Nascimento C.M., Ribeiro E.B., Lira F.S. (2015). Decaffeinated green tea extract rich in epigallocatechin-3-gallate prevents fatty liver disease by increased activities of mitochondrial respiratory chain complexes in diet-induced obesity mice. J. Nutr. Biochem..

[56] Ding Y., Sun X., Chen Y., Deng Y., Qian K. (2015). Epigallocatechin gallate attenuated non-alcoholic steatohepatitis induced by methionine- and choline-deficient diet. Eur. J. Pharmacol..

[57] Xiao J., Ho C.T., Liong E.C., Nanji A.A., Leung T.M., Lau T.Y., Fung M.L., Tipoe G.L. (2014). Epigallocatechin gallate attenuates fibrosis, oxidative stress, and inflammation in non-alcoholic fatty liver disease rat model through TGF/SMAD, PI3 K/Akt/FoxO1, and NF-kappa B pathways. Eur. J. Nutr..

[58] Kochi T., Shimizu M., Terakura D., Baba A., Ohno T., Kubota M., Shirakami Y., Tsurumi H., Tanaka T., Moriwaki H. (2014). Non-alcoholic steatohepatitis and preneoplastic lesions develop in the liver of obese and hypertensive rats: Suppressing effects of EGCG on the development of liver lesions. Cancer Lett..

[59] Du Y., Paglicawan L., Soomro S., Abunofal O., Baig S., Vanarsa K., Hicks J., Mohan C. (2021). Epigallocatechin-3-Gallate Dampens Non-Alcoholic Fatty Liver by Modulating Liver Function, Lipid Profile and Macrophage Polarization. Nutrients.

[60] Wu D., Liu Z., Wang Y., Zhang Q., Li J., Zhong P., Xie Z., Ji A., Li Y. (2021). Epigallocatechin-3-Gallate Alleviates High-Fat Diet-Induced Nonalcoholic Fatty Liver Disease via Inhibition of Apoptosis and Promotion of Autophagy through the ROS/MAPK Signaling Pathway. Oxidative Med. Cell. Longev..

[61] Ushiroda C., Naito Y., Takagi T., Uchiyama K., Mizushima K., Higashimura Y., Yasukawa Z., Okubo T., Inoue R., Honda A. (2019). Green tea polyphenol (epigallocatechin-3-gallate) improves gut dysbiosis and serum bile acids dysregulation in high-fat diet-fed mice. J. Clin. Biochem. Nutr..

[62] Xu K.-H., Yang D.-F., Liu M.-Y., Xu W., Li Y.-H., Xiao W.-J. (2024). Hepatoprotective effects and mechanisms of l-theanine and epigallocatechin gallate combined intervention in alcoholic fatty liver rats. J. Sci. Food Agric..

[63] Yun J.W., Kim Y.K., Lee B.S., Kim C.W., Hyun J.S., Baik J.H., Kim J.J., Kim B.H. (2007). Effect of dietary epigallocatechin-3-gallate on cytochrome P450 2E1-dependent alcoholic liver damage: Enhancement of fatty acid oxidation. Biosci. Biotechnol. Biochem..

[64] Ren Y., Deng F., Zhu H., Wan W., Ye J., Luo B. (2011). Effect of epigallocatechin-3-gallate on iron overload in mice with alcoholic liver disease. Mol. Biol. Rep..

[65] Megahed F.A.K., Zhou X., Sun P. (2020). The Interactions Between HBV and the Innate Immunity of Hepatocytes. Viruses.

[66] Salpini R., D’Anna S., Benedetti L., Piermatteo L., Gill U., Svicher V., Kennedy P.T.F. (2022). Hepatitis B virus DNA integration as a novel biomarker of hepatitis B virus-mediated pathogenetic properties and a barrier to the current strategies for hepatitis B virus cure. Front. Microbiol..

[67] Nassal M. (2015). HBV cccDNA: Viral persistence reservoir and key obstacle for a cure of chronic hepatitis B. Gut.

[68] Wu C.-C., Chen Y.-S., Cao L., Chen X.-W., Lu M.-J. (2018). Hepatitis B virus infection: Defective surface antigen expression and pathogenesis. World J. Gastroenterol..

[69] Mehmankhah M., Bhat R., Anvar M.S., Ali S., Alam A., Farooqui A., Amir F., Anwer A., Khan S., Azmi I. (2019). Structure-Guided Approach to Identify Potential Inhibitors of Large Envelope Protein to Prevent Hepatitis B Virus Infection. Oxidative Med. Cell. Longev..

[70] Cooper A., Paran N., Shaul Y. (2003). The earliest steps in hepatitis B virus infection. Biochim. Biophys. Acta (BBA)-Biomembr..

[71] Al-Bari A.A., Ito Y., Thomes P.G., Menon M.B., Garcia-Macia M., Fadel R., Stadlin A., Peake N., Faris M.E., Eid N. (2023). Emerging mechanistic insights of selective autophagy in hepatic diseases. Front. Pharmacol..

[72] Tian Y., Sir D., Kuo C.F., Ann D.K., Ou J.H. (2011). Autophagy required for hepatitis B virus replication in transgenic mice. J. Virol..

[73] Sir D., Tian Y., Chen W.L., Ann D.K., Yen T.S., Ou J.H. (2010). The early autophagic pathway is activated by hepatitis B virus and required for viral DNA replication. Proc. Natl. Acad. Sci. USA.

[74] Li J., Liu Y., Wang Z., Liu K., Wang Y., Liu J., Ding H., Yuan Z. (2011). Subversion of cellular autophagy machinery by hepatitis B virus for viral envelopment. J. Virol..

[75] Zhong L., Hu J., Shu W., Gao B., Xiong S. (2015). Epigallocatechin-3-gallate opposes HBV-induced incomplete autophagy by enhancing lysosomal acidification, which is unfavorable for HBV replication. Cell Death Dis..

[76] Petruzziello A., Marigliano S., Loquercio G., Cozzolino A., Cacciapuoti C. (2016). Global epidemiology of hepatitis C virus infection: An up-date of the distribution and circulation of hepatitis C virus genotypes. World J. Gastroenterol..

[77] Lingala S., Ghany M.G. (2015). Natural History of Hepatitis C. Gastroenterol. Clin. N. Am..

[78] Gemma S., Brogi S., Novellino E., Campiani G., Maga G., Brindisi M., Butini S. (2014). HCV-targeted Antivirals: Current Status and Future Challenges. Curr. Pharm. Des..

[79] Song J.M. (2018). Anti-infective potential of catechins and their derivatives against viral hepatitis. Clin. Exp. Vaccine Res..

[80] Fukazawa H., Suzuki T., Wakita T., Murakami Y. (2012). A cell-based, microplate colorimetric screen identifies 7,8-benzoflavone and green tea gallate catechins as inhibitors of the hepatitis C virus. Biol. Pharm. Bull..

[81] Mekky R.Y., El-Ekiaby N.M., Hamza M.T., Elemam N.M., El-Sayed M., Esmat G., Abdelaziz A.I. (2015). Mir-194 is a hepatocyte gate keeper hindering HCV entry through targeting CD81 receptor. J. Infect..

[82] Manns M.P., Lohse A.W., Vergani D. (2015). Autoimmune hepatitis—Update 2015. J. Hepatol..

[83] Hu Y., Gu J., Lin J., Wang Y., Yang F., Yin J., Yu Z., Wu S., Lv H., Ji X. (2021). (−)-Epigallocatechin-3-gallate (EGCG) modulates polarized macrophages to suppress M1 phenotype and promote M2 polarization in vitro and in vivo. J. Funct. Foods.

[84] Akhtar N., Haqqi T.M. (2011). Epigallocatechin-3-gallate suppresses the global interleukin-1beta-induced inflammatory response in human chondrocytes. Arthritis Res. Ther..

[85] Liu D., Zhang X., Jiang L., Guo Y., Zheng C. (2014). Epigallocatechin-3-gallate (EGCG) attenuates concanavalin A-induced hepatic injury in mice. Acta Histochem..

[86] Younossi Z.M., Koenig A.B., Abdelatif D., Fazel Y., Henry L., Wymer M. (2016). Global epidemiology of nonalcoholic fatty liver disease-Meta-analytic assessment of prevalence, incidence, and outcomes. Hepatology.

[87] Nikolova-Karakashian M. (2018). Alcoholic and non-alcoholic fatty liver disease: Focus on ceramide. Adv. Biol. Regul..

[88] Chen C., Liu Q., Liu L., Hu Y.Y., Feng Q. (2018). Potential Biological Effects of (−)-Epigallocatechin-3-gallate on the Treatment of Nonalcoholic Fatty Liver Disease. Mol. Nutr. Food Res..

[89] Neuschwander-Tetri B.A. (2017). Non-alcoholic fatty liver disease. BMC Med..

[90] Younossi Z.M. (2019). Non-alcoholic fatty liver disease—A global public health perspective. J. Hepatol..

[91] Pan M.H., Lai C.S., Tsai M.L., Ho C.T. (2014). Chemoprevention of nonalcoholic fatty liver disease by dietary natural compounds. Mol. Nutr. Food Res..

[92] Marin-Juez R., Jong-Raadsen S., Yang S., Spaink H.P. (2014). Hyperinsulinemia induces insulin resistance and immune suppression via Ptpn6/Shp1 in zebrafish. J. Endocrinol..

[93] Mu W., Cheng X.F., Liu Y., Lv Q.Z., Liu G.L., Zhang J.G., Li X.Y. (2018). Potential Nexus of Non-alcoholic Fatty Liver Disease and Type 2 Diabetes Mellitus: Insulin Resistance Between Hepatic and Peripheral Tissues. Front. Pharmacol..

[94] Yamamoto K., Ikeya T., Okuyama S., Fukuda K., Kobayashi D. (2021). The association between non-alcoholic fatty liver disease (with or without metabolic syndrome) and extrahepatic cancer development. J. Gastroenterol. Hepatol..

[95] Li X., Zhang Y., Zhao C., Zhang B., Peng B., Zhang Y., Wang J., Wang S. (2022). Positive effects of Epigallocatechin-3-gallate (EGCG) intervention on insulin resistance and gut microbial dysbiosis induced by bisphenol A. J. Funct. Foods.

[96] Luo K., Ma C., Xing S., An Y., Feng J., Dang H., Huang W., Qiao L., Cheng J., Xie L. (2020). White tea and its active polyphenols lower cholesterol through reduction of very-low-density lipoprotein production and induction of LDLR expression. Biomed. Pharmacother..

[97] Naito Y., Ushiroda C., Mizushima K., Inoue R., Yasukawa Z., Abe A., Takagi T. (2020). Epigallocatechin-3-gallate (EGCG) attenuates non-alcoholic fatty liver disease via modulating the interaction between gut microbiota and bile acids. J. Clin. Biochem. Nutr..

[98] Yu J., Marsh S., Hu J., Feng W., Wu C. (2016). Gut Microbiota and Metagenomic Advancement in Digestive Disease. Gastroenterol. Res. Pract..

[99] Takaki A., Kawai D., Yamamoto K. (2013). Multiple hits, including oxidative stress, as pathogenesis and treatment target in non-alcoholic steatohepatitis (NASH). Int. J. Mol. Sci..

[100] Yang X.H., Zhang B.L., Zhang X.M., Tong J.D., Gu Y.H., Guo L.L., Jin H.M. (2020). EGCG Attenuates Renal Damage via Reversing Klotho Hypermethylation in Diabetic db/db Mice and HK-2 Cells. Oxidative Med. Cell. Longev..

[101] Han J., Wang M., Jing X., Shi H., Ren M., Lou H. (2014). (−)-Epigallocatechin gallate protects against cerebral ischemia-induced oxidative stress via Nrf2/ARE signaling. Neurochem. Res..

[102] Dominguez F., Adler E., Garcia-Pavia P. (2024). Alcoholic cardiomyopathy: An update. Eur. Heart J..

[103] Thoudam T., Gao H., Jiang Y., Huda N., Yang Z., Ma J., Liangpunsakul S. (2024). Mitochondrial quality control in alcohol-associated liver disease. Hepatol. Commun..

[104] Luo P., Wang F., Wong N.K., Lv Y., Li X., Li M., Tipoe G.L., So K.F., Xu A., Chen S. (2020). Divergent Roles of Kupffer Cell TLR2/3 Signaling in Alcoholic Liver Disease and the Protective Role of EGCG. Cell. Mol. Gastroenterol. Hepatol..

[105] Villanueva A. (2019). Hepatocellular Carcinoma. N. Engl. J. Med..

[106] Xu J. (2018). Trends in Liver Cancer Mortality Among Adults Aged 25 and Over in the United States, 2000–2016. NCHS Data Brief.

[107] Zheng R., Qu C., Zhang S., Zeng H., Sun K., Gu X., Xia C., Yang Z., Li H., Wei W. (2018). Liver cancer incidence and mortality in China: Temporal trends and projections to 2030. Chin. J. Cancer Res..

[108] Craig A.J., von Felden J., Garcia-Lezana T., Sarcognato S., Villanueva A. (2020). Tumour evolution in hepatocellular carcinoma. Nat. Rev. Gastroenterol. Hepatol..

[109] Balogh J., Victor D., Asham E.H., Burroughs S.G., Boktour M., Saharia A., Li X., Ghobrial R.M., Monsour H.P. (2016). Hepatocellular carcinoma: A review. J. Hepatocell. Carcinoma.

[110] Niu L., Liu L., Yang S., Ren J., Lai P.B.S., Chen G.G. (2017). New insights into sorafenib resistance in hepatocellular carcinoma: Responsible mechanisms and promising strategies. Biochim. Biophys. Acta Rev. Cancer.

[111] Khiewkamrop P., Phunsomboon P., Richert L., Pekthong D., Srisawang P. (2018). Epistructured catechins, EGCG and EC facilitate apoptosis induction through targeting de novo lipogenesis pathway in HepG2 cells. Cancer Cell Int..

[112] Roomi M.W., Monterrey J.C., Kalinovsky T., Rath M., Niedzwiecki A. (2010). Comparative effects of EGCG, green tea and a nutrient mixture on the patterns of MMP-2 and MMP-9 expression in cancer cell lines. Oncol. Rep..

[113] Roomi M.W., Kalinovsky T., Bhanap B., Niedzwiecki A., Rath M. (2019). In Vitro Effect of Cytokines, Inducers, and Inhibitors on the Secretion of MMP-2 and MMP-9 in Hepatocarcinoma Cell Line SK-Hep-1. Integr. Cancer Ther..

[114] Zhang Y., Owusu L., Duan W., Jiang T., Zang S., Ahmed A., Xin Y. (2013). Anti-metastatic and differential effects on protein expression of epigallocatechin-3-gallate in HCCLM6 hepatocellular carcinoma cells. Int. J. Mol. Med..

[115] Zapf M.A., Kothari A.N., Weber C.E., Arffa M.L., Wai P.Y., Driver J., Gupta G.N., Kuo P.C., Mi Z. (2015). Green tea component epigallocatechin-3-gallate decreases expression of osteopontin via a decrease in mRNA half-life in cell lines of metastatic hepatocellular carcinoma. Surgery.

[116] Zhang G., Miura Y., Yagasaki K. (2000). Suppression of adhesion and invasion of hepatoma cells in culture by tea compounds through antioxidative activity. Cancer Lett..

[117] Jin J., Chang Y., Wei W., He Y.F., Hu S.S., Wang D., Wu Y.J. (2012). Prostanoid EP1 receptor as the target of (−)-epigallocatechin-3-gallate in suppressing hepatocellular carcinoma cells in vitro. Acta Pharmacol. Sin..

[118] Kang Q., Tong Y., Gowd V., Wang M., Chen F., Cheng K.W. (2021). Oral administration of EGCG solution equivalent to daily achievable dosages of regular tea drinkers effectively suppresses miR483-3p induced metastasis of hepatocellular carcinoma cells in mice. Food Funct..

[119] Ren T., Zhang H., Wang J., Zhu J., Jin M., Wu Y., Guo X., Ji L., Huang Q., Zhang H. (2017). MCU-dependent mitochondrial Ca^2+^ inhibits NAD^+^/SIRT3/SOD2 pathway to promote ROS production and metastasis of HCC cells. Oncogene.

[120] Kuo P.L., Lin C.C. (2003). Green tea constituent (−)-epigallocatechin-3-gallate inhibits Hep G2 cell proliferation and induces apoptosis through p53-dependent and Fas-mediated pathways. J. Biomed. Sci..

[121] Cho A.-R., Park W.-Y., Lee H.-J., Sim D.-Y., Im E., Park J.-E., Ahn C.-H., Shim B.-S., Kim S.-H. (2021). Antitumor Effect of Morusin via G1 Arrest and Antiglycolysis by AMPK Activation in Hepatocellular Cancer. Int. J. Mol. Sci..

[122] Shirakami Y., Shimizu M., Adachi S., Sakai H., Nakagawa T., Yasuda Y., Tsurumi H., Hara Y., Moriwaki H. (2009). (−)-Epigallocatechin gallate suppresses the growth of human hepatocellular carcinoma cells by inhibiting activation of the vascular endothelial growth factor-vascular endothelial growth factor receptor axis. Cancer Sci..

[123] Tang Y., Cao J., Cai Z., An H., Li Y., Peng Y., Chen N., Luo A., Tao H., Li K. (2020). Epigallocatechin gallate induces chemopreventive effects on rats with diethylnitrosamineinduced liver cancer via inhibition of cell division cycle 25A. Mol. Med. Rep..

[124] Caban M., Owczarek K., Chojnacka K., Lewandowska U. (2019). Overview of polyphenols and polyphenol-rich extracts as modulators of IGF-1, IGF-1R, and IGFBP expression in cancer diseases. J. Funct. Foods.

[125] Yin Y., Chen C., Chen J., Zhan R., Zhang Q., Xu X., Li D., Li M. (2017). Cell surface GRP78 facilitates hepatoma cells proliferation and migration by activating IGF-IR. Cell. Signal..

[126] Sur S., Pal D., Mandal S., Roy A., Panda C.K. (2016). Tea polyphenols epigallocatechin gallete and theaflavin restrict mouse liver carcinogenesis through modulation of self-renewal Wnt and hedgehog pathways. J. Nutr. Biochem..

[127] Gao F., Li M., Liu W.B., Zhou Z.S., Zhang R., Li J.L., Zhou K.C. (2015). Epigallocatechin gallate inhibits human tongue carcinoma cells via HK2-mediated glycolysis. Oncol. Rep..

[128] Li S., Wu L., Feng J., Li J., Liu T., Zhang R., Xu S., Cheng K., Zhou Y., Zhou S. (2016). In vitro and in vivo study of epigallocatechin-3-gallate-induced apoptosis in aerobic glycolytic hepatocellular carcinoma cells involving inhibition of phosphofructokinase activity. Sci. Rep..

[129] Tong J.L., Nie F., Ran Z.H., Pan C.Q., Xu X.T., Zhu M.M., Xiao S.D. (2009). Epigallocatechin gallate induces apoptosis in human hepatocellular carcinoma HepG2 cells via TGF/Smad signaling pathway. Zhonghua Zhong Liu Za Zhi.

[130] Shen X., Zhang Y., Feng Y., Zhang L., Li J., Xie Y.A., Luo X. (2014). Epigallocatechin-3-gallate inhibits cell growth, induces apoptosis and causes S phase arrest in hepatocellular carcinoma by suppressing the AKT pathway. Int. J. Oncol..

[131] Tsang W.P., Kwok T.T. (2010). Epigallocatechin gallate up-regulation of miR-16 and induction of apoptosis in human cancer cells. J. Nutr. Biochem..

[132] Jiang X., Wang J., Deng X., Xiong F., Zhang S., Gong Z., Li X., Cao K., Deng H., He Y. (2020). The role of microenvironment in tumor angiogenesis. J. Exp. Clin. Cancer Res..

[133] Liao Z.-H., Zhu H.-Q., Chen Y.-Y., Chen R.-L., Fu L.-X., Li L., Zhou H., Zhou J.-L., Liang G. (2020). The epigallocatechin gallate derivative Y 6 inhibits human hepatocellular carcinoma by inhibiting angiogenesis in MAPK/ERK1/2 and PI3K/AKT/HIF-1α/VEGF dependent pathways. J. Ethnopharmacol..

[134] Hashimoto O., Nakamura A., Nakamura T., Iwamoto H., Hiroshi M., Inoue K., Torimura T., Ueno T., Sata M. (2014). Methylated-(3″)-epigallocatechin gallate analog suppresses tumor growth in Huh7 hepatoma cells via inhibition of angiogenesis. Nutr. Cancer.

[135] Yang C.S., Chen T., Ho C.-T. (2022). Redox and Other Biological Activities of Tea Catechins That May Affect Health: Mechanisms and Unresolved Issues. J. Agric. Food Chem..

[136] Nikoo M., Regenstein J.M., Ahmadi Gavlighi H. (2018). Antioxidant and Antimicrobial Activities of (−)-Epigallocatechin-3-gallate (EGCG) and its Potential to Preserve the Quality and Safety of Foods. Compr. Rev. Food Sci. Food Saf..

[137] Dekant W., Fujii K., Shibata E., Morita O., Shimotoyodome A. (2017). Safety assessment of green tea based beverages and dried green tea extracts as nutritional supplements. Toxicol. Lett..

[138] Shiha G., Soliman R., Elbasiony M., Darwish N.H.E., Mousa S.A. (2019). Addition of Epigallocatechin Gallate 400 mg to Sofosbuvir 400 mg + Daclatisvir 60 mg with or Without Ribavirin in Treatment of Patients with Chronic Hepatitis C Improves the Safety Profile: A Pilot Study. Sci. Rep..

[139] Shiha G., Soliman R., Elbasiony M., Darwish N.H.E., Mousa S.A. (2021). Novel combined single dose anti-hepatitis C therapy: A pilot study. Sci. Rep..

[140] Zhu Z., Wang Y., Liu Z., Wang F., Zhao Q. (2012). Inhibitory effects of epigallocatechin-3-gallate on cell proliferation and the expression of HIF-1α and P-gp in the human pancreatic carcinoma cell line PANC-1. Oncol. Rep..

[141] Chen L., Ye H.L., Zhang G., Yao W.M., Chen X.Z., Zhang F.C., Liang G. (2014). Autophagy inhibition contributes to the synergistic interaction between EGCG and doxorubicin to kill the hepatoma Hep3B cells. PLoS ONE.

[142] Wang H., Jiang H., Zhou M., Xu Z., Liu S., Shi B., Yao X., Yao M., Gu J., Li Z. (2009). Epidermal growth factor receptor vIII enhances tumorigenicity and resistance to 5-fluorouracil in human hepatocellular carcinoma. Cancer Lett..

[143] Yang X.W., Wang X.L., Cao L.Q., Jiang X.F., Peng H.P., Lin S.M., Xue P., Chen D. (2012). Green tea polyphenol epigallocatechin-3-gallate enhances 5-fluorouracil-induced cell growth inhibition of hepatocellular carcinoma cells. Hepatol. Res..

[144] Sabry D., Abdelaleem O.O., El Amin Ali A.M., Mohammed R.A., Abdel-Hameed N.D., Hassouna A., Khalifa W.A. (2019). Anti-proliferative and anti-apoptotic potential effects of epigallocatechin-3-gallate and/or metformin on hepatocellular carcinoma cells: In vitro study. Mol. Biol. Rep..

[145] Yang H., Wang M., Sun H., Zhu S., Jin J. (2019). Synergetic Effect of EP1 Receptor Antagonist and (−)-Epigallocatechin-3-gallate in Hepatocellular Carcinoma. Pharmacology.

[146] Abou El Naga R.N., Azab S.S., El-Demerdash E., Shaarawy S., El-Merzabani M., Ammar E.M. (2013). Sensitization of TRAIL-induced apoptosis in human hepatocellular carcinoma HepG2 cells by phytochemicals. Life Sci..

